# Dynamic antibody response in SARS-CoV-2 infected patients and COVID-19 vaccine recipients alongside vaccine effectiveness in comorbid and multimorbid groups

**DOI:** 10.1016/j.heliyon.2023.e16349

**Published:** 2023-05-20

**Authors:** Depro Das, Fahmida Khanam Raha, Khondekar Mustaq Adnan, Md Rubayet Siraj, Mariam Jamila Shapla, Farzana Shumy, Md Emdadul Haque, Monwar Hasanat Khan, Susmita Sanyal, Md Ismail Hosen, AHM Nurun Nabi, Mousumi Sanyal, Sajib Chakraborty, Md Zahid Amin

**Affiliations:** a Systems Cell-Signalling Laboratory, Department of Biochemistry and Molecular Biology, University of Dhaka, Dhaka 1000, Bangladesh; b Central Police Hospital, Dhaka, Bangladesh; c Square Hospitals Ltd, Dhaka, Bangladesh; d Clinical Biochemistry and Translational Medicine Laboratory, Department of Biochemistry and Molecular Biology, University of Dhaka, Dhaka 1000, Bangladesh; e Laboratory of Population Genetics, Department of Biochemistry and Molecular Biology, University of Dhaka, Dhaka 1000, Bangladesh

**Keywords:** IgG, Antibody response, SARS-CoV-2, COVID-19, Multimorbidity, Kidney disease, Diabetes, Bangladesh

## Abstract

**Objectives:**

Underlying medical conditions are critical risk factors for COVID-19 susceptibility and its rapid clinical manifestation. Therefore, the preexisting burden of non-communicable diseases (NCDs) makes the preparedness for COVID-19 more challenging for low- and middle-income countries (LMICs). These countries have relied on vaccination campaigns as an effective measure to tackle COVID-19. In this study, we investigated the impact of comorbidities on humoral antibody responses against the specific receptor-binding domain (RBD) of SARS-CoV2.

**Methods:**

A total of 1005 patients were selected for the SARS-CoV-2 specific immunoglobulin G (IgG1, IgG2, IgG3, and IgG4 subclasses) and total antibody (TAb) tests (IgG and IgM), of which 912 serum samples were ultimately selected based on the specimen cutoff analyte value. Patients with multimorbidity (N = 60) were recruited for follow-up studies from the initial cohort, and their immune response (IgG and TAb) was measured at multiple time points after the second dose of vaccination. Siemens Dimension Vista SARS-CoV-2 IgG (CV2G) and SARS-CoV-2 TAb assay (CV2T) were used to carry out the serology test.

**Results:**

Out of a total of 912 participants, vaccinated individuals (N = 711) had detectable antibody responses up to 7–8 months. The synergistic effect of natural infection and vaccine response was also studied. Participants with breakthrough infections (N = 49) mounted a greater antibody response compared to individuals with normal vaccination response (N = 397) and those who were naturally infected before receiving the second dose of vaccine (N = 132). Investigation of the impact of comorbidities revealed that diabetes mellitus (DM) (N = 117) and kidney disease (N = 50) had a significant negative impact on the decline of the humoral antibody response against SARS-CoV-2. IgG and TAb declined more rapidly in diabetic and kidney disease patients compared to the other four comorbid groups. Follow-up studies demonstrated that antibody response rapidly declined within 4 months after receiving the second dose.

**Conclusion:**

The generalized immunization schedule for COVID-19 needs to be adjusted for high-risk comorbid groups, and a booster dose must be administered early within 4 months after receiving the second dose.

## 1 Introduction

COVID-19 is predominantly an airborne respiratory viral disease that is caused by the infectious Severe acute respiratory syndrome coronavirus 2 (SARS-CoV-2). Since its early emergence at late December 2019, in Wuhan, China, the novel form of the virus has affected over 216 countries globally [1]. Bangladesh also has been a major sufferer to share its casualties alongside the rest of the world. The country detected its first COVID-19 cases on March 8, 2020, and reported its first death due to COVID-19 illness on March 18, 2020. Almost 29,127 COVID-19 related nationwide deaths have been documented since the pandemic began. A 70-year-old man with multiple chronic illnesses, such as diabetes, kidney diseases, hypertension, and heart disease, was the first victim [2,3]. Crucial epidemiologic risk factors associated with high COVID-19 disease severity include older age (≥60) and male gender, and the highest susceptibility was observed in Black and South Asian ethnic groups [4]. Irrespective of these demographic factors, any individual with underlying medical conditions, such as diabetes mellitus (DM), obesity (BMI ≥40), hypertension, asthma, cardiovascular disease, kidney disease, cancer, or autoimmune disease, is classified as a high-risk group for COVID-19 related mortality [5,6]. Bangladesh already shares a heavy global burden of these non-communicable diseases (NCDs) which account for almost 59% of total deaths in the country per year [7]. In these medically compromised patients, the preexisting conditions can exacerbate the pathophysiology of the COVID-19 infection making the person highly vulnerable. Inadequate supply of safety equipment, low number of tests, and lack of skilled human resources are some of the major constraints in most low- and middle-income countries (LMICs) for tackling COVID-19 [8]. Most LMICs rely on the COVAX (COVID-19 Vaccines Global Access) facility to import and allocate vaccines [9]. According to the Directorate General of Health Services (DGHS), Bangladesh has administered vaccines manufactured by 6 primary developers, including AstraZeneca (ChAdOx1), Pfizer-BioNTech (BNT162b2), Moderna (mRNA-1273), Sinovac (CZ02), and Sinopharm (BBIBP-CorV) [10]. Regardless of the vaccine type, all of them possess the potential to rapidly induce protective immunity against SARS-CoV-2. Vaccine recipients evoke a more potent immune response and confer humoral and cell-mediated immunity, which are two different forms of the adaptive immune system. T cell-mediated cellular immunity has been shown to restrict the viral spread and resolve viral infection to reduce disease burden. However, SARS-CoV-2 specific antibodies (IgA and IgG) are of primary importance, as they are the initial mediators of humoral immunity. These humoral immunoglobulins robustly neutralize the virus, ensure protection against secondary infection, and are easily detectable within several months of vaccination [11,12]. While this is the primary incentive for vaccine development, different cohort studies have reported that protective immune response persists over 6–8 months after receiving at least one dose of COVID-19 vaccine [[13], [14], [15], [16]].

The Government of Bangladesh has taken extensive measures to conduct mass vaccination campaigns to provide early protection against SARS-CoV-2. The country has administered 357,087,045 doses of COVID-19 vaccines so far with 80.31% (136,784,348) people receiving their second dose. Only 49.11% (67,178,742) of the people receiving the second dose were administered with the first booster dose, leaving a huge population behind [10]. To vaccinate another 10% of the population, at least 225 days are required with a daily vaccination rate of 145,282 doses [17]. This scenario puts the elderly population as well as different comorbid groups in the high-risk category. Considering these circumstances in terms of Bangladesh, no studies have been reported to showcase any clear correlation of SARS-CoV-2 specific antibody response within a heterogeneous population to reach critical assumptions all at once.

In this multi-dimensional study, we have stratified our total population based on vaccination status and previous records of SARS-CoV-2 exposure and explained the relationships of antibody response between age, gender, and prevalent comorbidities. We have also investigated the synergistic effect of infection-acquired immunity on vaccinated participants. Lastly, high-risk comorbid groups were identified and their temporal variation in antibody response was observed for three-time points.

## 2 Materials and methods

### 2.1 Characteristics and classification of study subjects

A total of 1005 participants aged ≥18 years from the general population were enrolled in the study, which included a SARS-CoV-2 anti-spike immunoglobulin G (IgG1, IgG2, IgG3, and IgG4 subclasses) and total antibody (TAb) test (IgG and IgM). The serum samples were collected for a serology test after obtaining signed informed consent from all participants, and refusal to participate in the study was the primary exclusion criteria. The manufacturer-specified specimen cutoff analyte value was used as the second exclusion criteria, with this value being different for IgG and TAb [18]. As a result, false-positives were discarded and only true-positive results were retained for further analysis.

All true-positive participants were classified into three groups according to their vaccination status and previous exposure to SARS-CoV-2. The classified groups were designated as vaccinated, unvaccinated, and naturally infected. Participants who received at least the first dose of the COVID-19 vaccine (Pfizer, Moderna or AstraZeneca) were categorized as the vaccinated group. The unvaccinated group included asymptomatic individuals who did not receive any COVID-19 vaccine. The population of this group included the first-line hospital personnel who came in close contact with hospitalized COVID-19 patients. The asymptomatic status of the unvaccinated participants was established by laboratory-confirmed PCR test reports and these individuals suffered no prior infection according to their oral testimony. The naturally infected group incorporated the participants who suffered symptomatic COVID-19 infection and did not receive any COVID-19 vaccines according to oral testimony. The status for natural infection was confirmed by RT-PCR test reports.

The vaccinated group was further stratified into three categories. These categories were denoted as: pre-infected, post-infected, and uninfected. The pre-infected volunteers suffered symptomatic infection before receiving the second dose of COVID-19 vaccine. The post-infected volunteers showed symptomatic infection after the second dose of COVID-19 vaccine was administered (breakthrough infection). In both of these subgroups, the symptomatic infections associated with each patient were verified by RT-PCR testing. On the contrary, the uninfected subjects were orally confirmed to be non-symptomatic and non-infected before or after receiving the second dose. These volunteers represented a subset of the normally vaccinated participants.

**Fig. 1:**
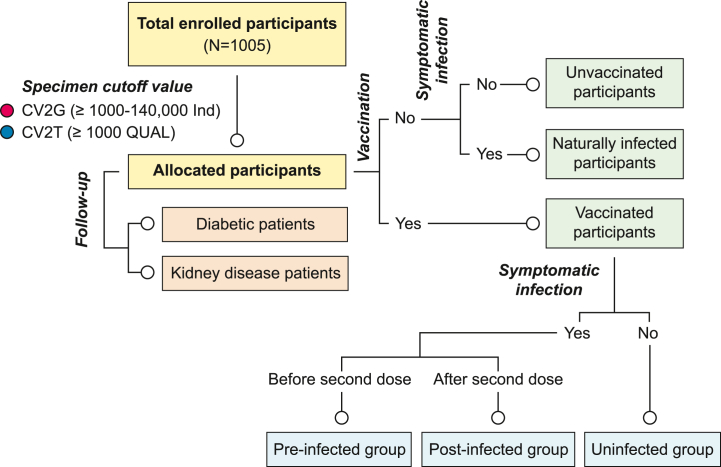
A flow diagram representing the characterization and classification of the study subjects.

Antibody response in immunocompromised patients with diabetes mellitus and kidney disease was the primary focus of this study. To further examine the dynamics of the antibody response across 3-time points, 31 patients primarily diagnosed with diabetes mellitus and 29 patients with kidney disease were selected from the initial true-positive participants and randomly assigned for follow-up studies. Fig. 1 depicts the classification of study participants.

### 2.2 Laboratory measurements

Siemens Dimension Vista SARS-CoV-2 IgG (CV2G) and SARS-CoV-2 TAb (CV2T) [19,20] assays were performed using the Dimension EXL 200 Integrated Chemistry System (Siemens Healthcare Diagnostics, Delaware, USA). The serologic assay was approved and authorized by the U.S. Food and Drug Administration (FDA) under an emergency use authorization (EUA) at the time of the study. This technique is a sandwich chemiluminescent immunoassay based on Luminescent Oxygen Channeling Immunoassay (LOCI) module. The test was designed to measure the antibodies directed against the S1 region of the receptor-binding domain (RBD) of SARS-CoV-2. LOCI reagents and SARS-CoV-2 specific antibody sandwiches are illuminated at a wavelength of 680 nm. The resulting signal after the chemiluminescent reaction is measured at 612 nm, which is a direct function of the antibody concentrations. Semi-quantitative mode or default mode was used to measure IgG level and the unit is quantified in Ind. The qualitative mode was used to measure the TAb in the QUAL unit [20]. Antibody levels ≥1000-140,000 Ind and ≥1000 QUAL were interpreted as positive for IgG and TAb, respectively [18]. According to the manufacturer, 1000 QUAL is equivalent to 12 BAU/ml for CV2T and 1000 Ind represents 17 BAU/ml for CV2G [20].

### 2.3 Statistical analysis

Shapiro-Wilks test was used to check for normality of the data. Descriptive statistics were used to summarize the data, categorical variables were represented as percentages, and continuous variables were described using the median, interquartile range (IQR), and 95% confidence interval (CI). Kruskal–Wallis H test with Dunn's posthoc alongside Bonferroni adjustment was used to calculate significance level and pairwise comparison between three main groups, three subgroups of vaccinated individuals, different comorbidities, and different time points for vaccinated individuals after receiving second dose of vaccine. Mann-Whitney *U* test was used to determine the pairwise comparisons between gender groups. Spearman Correlation test was used to assess the significance of antibody levels in two age groups (<60, ≥60 years) as well as measure the relationship of antibody levels in three vaccinated subgroups and each comorbid group after receiving second dose of vaccine. Data normalization and computation of Z-scores were done using R programming language [21]. Z-score was calculated from normalized data on the basis of antibody response for 3-time points (t1, t2, t3). Afterwards, hierarchical clustering was performed using the ‘hclust’ function, and Euclidean distance was used to group similar patient attributes based on the Z-value [22]. Data manipulation, visualization, and other exploratory data analysis were performed using R programming language and RStudio (version 4.1.0) [23]. A P-value of 0.05 or lower (P-value ≤0.05) was considered statistically significant throughout the entire study.

## 3 Results

### 3.1 Impact of diverse comorbidities on antibody response across vaccinated, unvaccinated, and naturally infected groups

**Fig. 2:**
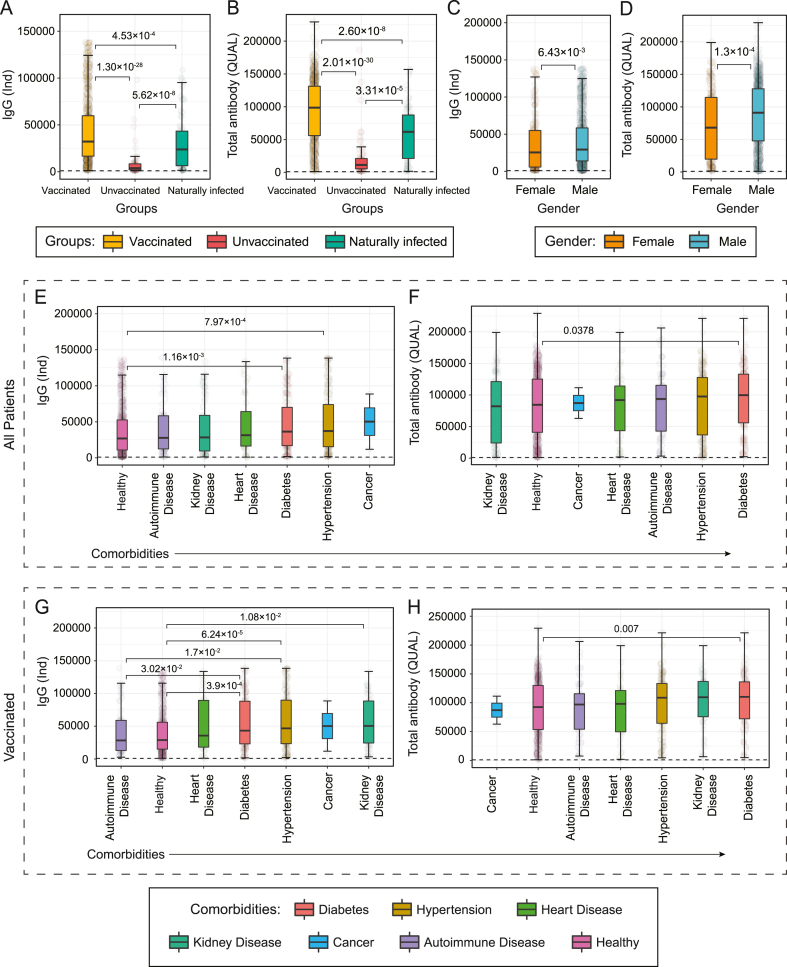
**IgG and TAb response across major patient groups with respect to gender and comorbidities.** Comparison of IgG (A) and TAb (B) response against S1-RBD of SARS-CoV-2 between major three groups (Vaccinated, Unvaccinated, and Naturally infected) and significant association of IgG (C) and TAb (D) response between male and female in total population. SARS-CoV-2 specific significant IgG (E) and TAb (F) response in all patients and IgG (G) and TAb (H) response in the Vaccinated group, categorized by different comorbidities (Diabetes, Hypertension, Heart Disease, Kidney Disease, Cancer, Autoimmune Disease). The box plots represent 25th to 75th percentiles, the horizontal line represents the median, and the whiskers are calculated by hinge ±1.5 × IQR. Each dot indicates the level of antibodies of one participant. P-values were calculated using Kruskal–Wallis H test with Dunn's posthoc alongside Bonferroni adjustment. Mann-Whitney *U* test was used for pairwise comparisons between two gender groups. The dashed line represents the specimen cutoff value.

Among the enrolled study participants (N = 1005), 912 individuals were selected through the two exclusion criteria and comprised the initial cohort. Afterwards, 771 individuals (84.54%) were classified into the vaccinated group, 73 individuals (8%) into the unvaccinated group, and 68 individuals (7.46%) into the naturally infected group (Supplementary Table 1). As expected, SARS-CoV-2 specific IgG (Fig. 2A) and TAb (Fig. 2B) responses were significantly higher in the vaccinated group compared to other groups. Antibody response (IgG and TAb) in the unvaccinated group was the lowest. IgG and TAb responses were found to be higher in the naturally infected group compared to the unvaccinated group, but lower than the vaccinated group. To assess the gender-wise vaccine response, we studied the IgG and TAb levels in male and female participants. Within our total population, 719 (78.84%) participants were male, and 193 (21.16%) were female. The median level of IgG antibodies was higher in male than in female participants (p = 6.43 × × 10^−3^) (Fig. 2C). Similarly, the TAb response was 1.3 times higher in male than in female participants (p = 1.3 × 10^−4^) (Fig. 2D). The vaccinated group showed a similar pattern of immune response (Supplementary Figs. 1A and 1B). Comparisons of the median IgG and TAb responses revealed that males have a stronger IgG response and females have a stronger TAb response in both the unvaccinated (Supplementary Figs. 1C and 1D) and naturally infected (Supplementary Figs. 1E and 1F) groups. IgG and TAb levels were not significantly different between male and female subjects. Previously, it has been reported that individuals aged ≥60 are at greater risk of developing severe symptoms upon COVID-19 infection [[24], [25], [26]]. To investigate the association of age with vaccine response, we stratified the individuals (N=906) aged <60 (N=827) (91.28%) and classified them as adults, whereas individuals aged ≥60 (N=79) (8.72%) were stratified as elderly people. The median IgG and TAb levels were not significant but were observed to be higher in adults across the total participants (Supplementary Figs. 1G and 1H). The trend was opposite in the vaccinated (Supplementary Figs. 1I and 1J) and unvaccinated (Supplementary Fig. 1K, 1L) groups. Adults in the naturally infected group had a higher IgG response, while the elders had a higher TAb response (Supplementary Fig. 1M, 1N). In summary, the results showed that COVID-19 vaccination contributes to the higher levels of IgG and TAb compared to naturally infected subjects. Furthermore, we did not observe a significant influence of age and gender on IgG and TAb levels regardless of vaccination, asymptomatic infection, and natural infection.

The majority (64.14%) of the participants were healthy individuals (N=585); the remaining subjects had previously diagnosed comorbidities, a number of which were present in more than one patient. Hypertension was the most prominent comorbidity (N=168) (18.42%), followed by diabetes mellitus (DM) (N=152) (16.67%), kidney disease (N=73) (8%), autoimmune disorder (N=73) (8%), heart disease (N=40) (4.39%), and cancer (N=2) (0.22%) (Supplementary Table 2). In order to assess the impact of these diverse comorbidities on the antibody response, patients were stratified based on the first-diagnosed comorbidity. The lowest IgG level was observed in healthy individuals, followed by the comorbid groups, including autoimmune disease, kidney disease, heart disease, diabetes, hypertension, and cancer patients. Diabetic (p = 1.16 × 10^−3^) and hypertensive (p = 7.97 × 10^−4^) groups were significantly associated with the healthy population (Fig. 2E). Patients with kidney disease had the least TAb levels, preceded by healthy individuals and those with cancer, heart disease, autoimmune disease, hypertension, and diabetes, with only a significant association between diabetes and the healthy group (p = 0.0378) (Fig. 2F). The vaccinated population responded least strongly for IgG (Fig. 2G) and TAb (Fig. 2H) to autoimmune disease and cancer and most strongly to kidney disease and diabetes. The comorbid groups and the healthy population were found to have several significant associations. However, both IgG (p = 3.9 × 10^−4^) and TAb (p = 0.007) levels were found to be associated between the diabetic and healthy groups. A similar scenario was also noticed in the unvaccinated (Supplementary Figs. 2A and 2B) and naturally infected groups (Supplementary Figs. 2C and 2D) where the IgG and TAb responses were comparatively lower in the healthy subjects than in the comorbid patients. Supplementary Table 3 depicts the findings of pairwise comparisons for IgG and TAb response across all comorbid patient groups.

### 3.2 The dynamic decline of IgG and TAb levels in the vaccinated group

**Fig. 3:**
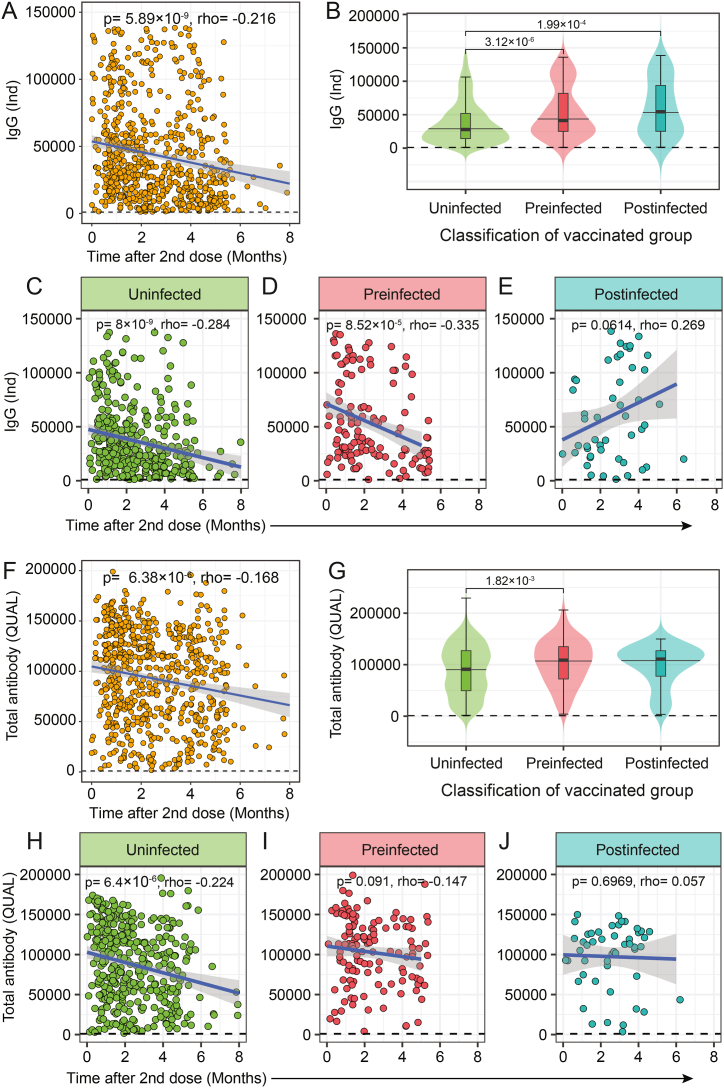
**The trends in IgG and TAb response over time in uninfected, pre-infected, post-infected subjects.** Scatter-plots for SARS-CoV-2 specific IgG (A) and TAb (F) response in the Vaccinated group at 8 different time points (0–8 months) after receiving second dose of vaccine. Significant association of IgG (B) and TAb (G) levels between three vaccinated subgroups. Discrete trends in IgG response in uninfected (C), pre-infected (D), and post-infected (E) individuals alongside TAb response in uninfected (H), pre-infected (I), and post-infected (J) individuals. The box plots represent 25th to 75th percentiles. The horizontal line represents the median, and the whiskers are calculated by hinge ±1.5 × IQR. Spearman Correlation Coefficient test was used to determine the rho (ρ) and P-values in the vaccinated group and the three subgroups. Kruskal–Wallis H test with Dunn's posthoc alongside Bonferroni adjustment was used for pairwise comparisons between the three subgroups. The dashed line represents the specimen cutoff value.

For 711 vaccinated participants (83.83% male and 16.17% female) out of the total number of vaccinated subjects (N = 771), the IgG and TAb measurements were conducted at a single time point per subject within 0–8 months after the second dose of vaccination due to the variability between the vaccination schedule and the antibody measurement time points (0–8 months). The median age of these participants was 36 years (IQR = 28–46, 95%CI = 37.34–39.12). The strongest IgG and TAb responses were observed in the 1st month after receiving the second dose. Both IgG and TAb titers gradually dropped over the next few months, reaching a low point after the 6th month, with further declines occurring in months 7 and 8 (Supplementary Figs. 3A and 3B). Correlation analysis revealed that IgG (Fig. 3A) and TAb (Fig. 3F) levels exhibited a strong negative correlation (IgG: rho = −0.216, TAb: rho = −0.168), with respect to time. The summary statistic and pairwise comparisons between definite time points (0–8 months) are provided in Supplementary Table 4 and Supplementary Table 5.

To further examine the additive effect of natural infection and vaccination on the antibody responses, a subset of the vaccinated participants (N = 578) was categorized into three groups (pre-infected, post-infected, and uninfected). Of these participants, 132 (22.84%) were pre-infected, 49 (8.48%) were post-infected, and 397 (68.68%) were uninfected. The IgG (Fig. 3B) and TAb (Fig. 3G) levels were lowest in the uninfected population, followed by the pre-infected group, while the post-infected subjects mounted a stronger immune response. Compared to the uninfected population, the IgG response was 1.5-fold higher in pre-infected patients and 1.8-fold higher in post-infected subjects, while the TAb response was 1.2-fold higher in both groups. The IgG and TAb levels declined in both uninfected (IgG: rho = −0.284, TAb: rho = −0.224) (Fig. 3C and H) and pre-infected (IgG: rho = −0.335, TAb: rho = −0.147) groups (Fig. 3D and I). In contrast, a modest increase in IgG and TAb levels was observed in the post-infected group (IgG: rho = 0.269, TAb: rho = 0.057) (Fig. 3E and J). The rate of IgG decay was significant in pre-infected (p = 8.52 × 10^−5^) and uninfected groups (p = 8 × 10^−9^). The decay of TAb in uninfected subjects also remained significant (p = 6.4 × 10^−6^). The summary statistic and pairwise comparisons between these three vaccinated sub-groups are presented in Supplementary Table 6. The IgG levels in pre-infected subjects had markedly decreased after 6 months, implying that the SARS-CoV-2 specific antibody response may not be sufficient to render protection against COVID-19 without a booster dose. The uninfected subcategory served as a representative class of the primary vaccinated group. Therefore, the trend of declining antibodies in the uninfected group for 8 months validated the prior finding.

### 3.3 Effect of diverse comorbidities on the decline of IgG and TAb response in vaccinated patients

**Fig. 4:**
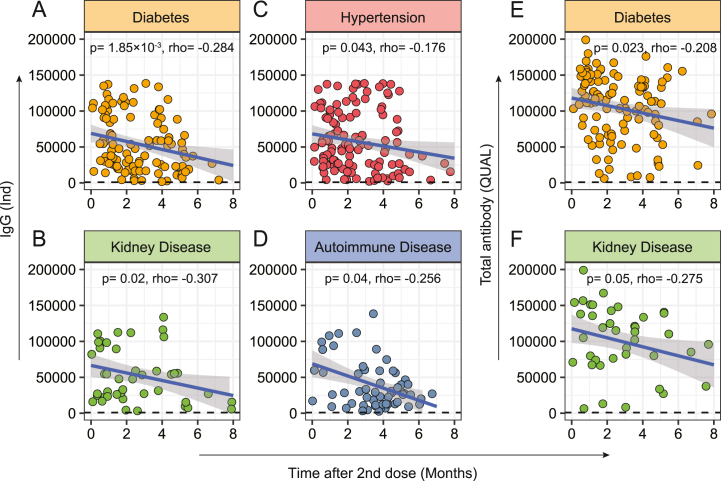
**The decline of IgG and TAb response over time in patients with comorbidities.** Scatter-plot for SARS-CoV-2 specific IgG and TAb response in vaccinated individuals with different comorbidities. IgG response in patients with diabetes (A), kidney disease (B), hypertension (C), and autoimmune disease (D) alongside TAb response in patients with diabetes (E), and kidney disease (F). Each dot represents the antibody level after second dose of vaccine in an individual patient. P-values and rho (ρ) were determined using the Spearman Correlation Coefficient test. The dashed line represents the specimen cutoff value.

IgG and TAb levels steadily decreased in each comorbid group, including, diabetes (N = 117) (Fig. 4A and E), kidney disease (N = 50) (Fig. 4B and F), hypertension (N = 131) (Fig. 4C, Supplementary Fig. 4C), autoimmune disease (N = 59) (Fig. 4D, Supplementary Fig. 4D), heart disease (N = 31) (Supplementary Figs. 4A and 4E), and cancer (N = 2) (Supplementary Figs. 4B and 4F), within 8 months after the second dose of vaccination. Significant decline of IgG levels as evident by the strong negative correlation was observed for diabetic (Fig. 4A), kidney disease (Fig. 4B), hypertension (Fig. 4C), and autoimmune disease patients (Fig. 4D). Meanwhile, the decline of the TAb response was significant for diabetic (Fig. 4E) and kidney disease patients (Fig. 4F). Altogether, the results hinted toward the possibility that the significant rapid decline of IgG and TAb levels in diabetes and kidney disease patients pose a significant challenge for maintaining a higher SARS-CoV-2 specific antibody titer.

### 3.4 Role of multimorbidity in the patient-specific temporal variation of IgG and TAb levels

For sub-cohorts of the diabetic (N = 31) and kidney disease patients (N = 29), antibody measurements were conducted at three time points (t1, t2, and t3) for each patient. Time point t1 represents the period immediately after the second dose, while time points t2 and t3 correspond, respectively, to the 3rd and 4th month after the second dose of vaccination. Follow-up patients were designated as vaccinated, unvaccinated, and naturally infected based on their initial status at time point t1.

**Fig. 5:**
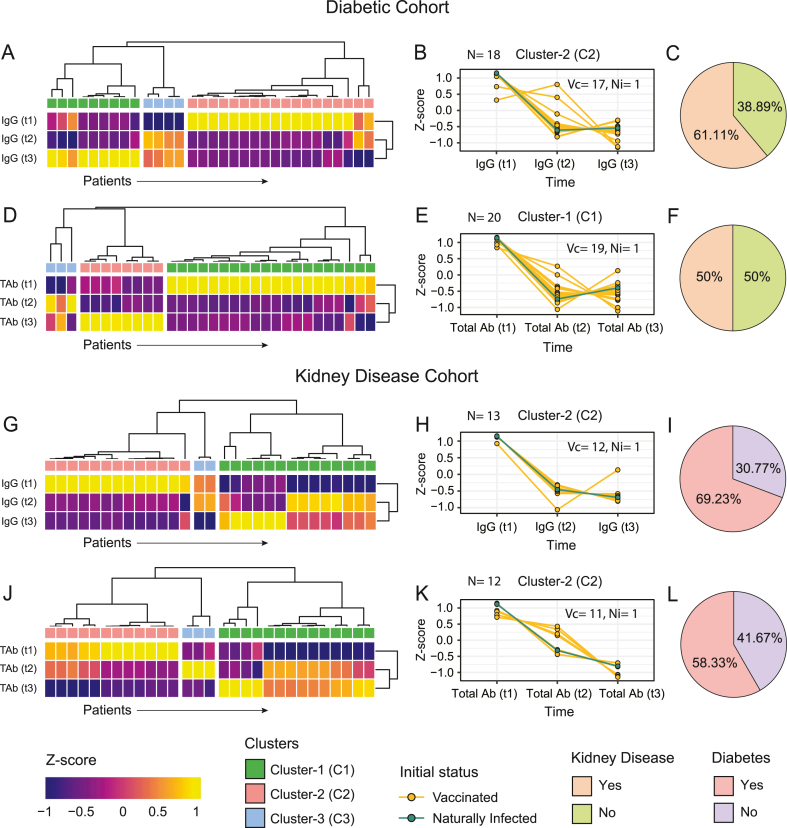
**Patient-specific temporal variation of IgG and TAb levels in patients with Diabetes and Kidney Disease in factual clusters.** Dynamic SARS-CoV-2 antibody response at three time points in patients with diabetes mellitus (N = 31) and kidney disease (N = 29). Hierarchical cluster analysis of IgG (A) and TAb (D) response (Z-scored) in individual patients is displayed as heatmaps for diabetes alongside IgG (G) and TAb (J) response (Z-scored) displayed as heatmaps for kidney disease for three different time points (t1, t2, and t3). The profile plots represent the factual trends of IgG (B) and TAb (E) response in diabetic patients for C2 and C1 alongside IgG (H) and TAb (K) response in kidney disease patients for C2. The percentage of diabetic patients diagnosed withs secondary kidney disease for IgG response in C2 (C) and TAb response in C1 (F) alongside kidney disease patients diagnosed with secondary diabetes for IgG response in C2 (I) and TAb response in C2 (L).

Patients in this diabetic sub-cohort had a median age of 52 years (IQR = 42–58, 95%CI = 47.59–56.79). Hierarchical clustering algorithm was employed on the Z-scored IgG and TAb levels to explore the dynamic change in antibody response. IgG (Fig. 5A) and TAb (Fig. 5D) levels formed three clusters – denoted as C1, C2, and C3. The majority of the diabetic patients (N = 18, 58.06%) were clustered in C2 based on the IgG response, in which all patients were vaccinated (54.84%) except one who was naturally infected (3.22%). IgG levels were substantially higher at t1, gradually decreased at t2 within 3 months, and reached a steady level at t3 within another 1 month (Fig. 5B). A similar trend of antibody decay was also observed for TAb in C1, which included 20 patients (64.52%), among whom 19 (61.29%) were vaccinated and 1 (3.22%) was naturally infected (Fig. 5E), according to their initial status at t1. Therefore, these two clusters represented the factual clusters. IgG response for C1 (Supplementary Fig. 5A) comprised of 9 vaccinated patients (29.03%) and TAb response for C2 (Supplementary Fig. 5C) comprised of 8 vaccinated patients (25.81%). The initial IgG levels at t1 showed higher variability, decreased at t2, and showed an elevation at t3. C3 for IgG (N = 4) and TAb (N = 3) responses represents the smallest cluster with only 12.9% and 9.68% of vaccinated patients, respectively. The initial IgG levels of C3 patients were very low at t1, significantly increased at t2, and decreased to a steady level at t3 (Supplementary Figs. 5E and 5G). The medical records of these vaccinated patients revealed that they had received a booster dose in the interim follow-up, which accounts for the sudden and dramatic increase in antibody levels seen in these adulterated clusters.

Akin to the previous analysis, the sub-cohort of kidney disease patients were divided into 3 clusters based on Z-scored IgG (Fig. 5G) and TAb (Fig. 5J) responses. The patients had a median age of 54 years (IQR = 44–64, 95%CI = 48.27–56.01). In accordance with the IgG response, 13 patients (44.83%) were grouped in C2, in which 12 (41.38%) were vaccinated, and 1 (3.45%) was naturally infected (Fig. 5H). For TAb response, C2 included 12 patients (41.38%). Among them, 11 patients (37.93%) were vaccinated, and 1 patient (3.45%) was naturally infected (Fig. 5K). In both of these factual clusters, the initial IgG was very high at t1 and progressively decreased at t2 and t3 within 4 months. C1 for both IgG (Supplementary Fig. 5I) and TAb (Supplementary Fig. 5K) responses included 14 patients (48.27%), of whom 7 (24.14%) were vaccinated and another 7 (24.14%) were unvaccinated, according to their initial status at t1. The initial antibody responses (IgG and TAb) were lower at t1 and remarkably increased at t2 and t3 within 4 months. Only 2 vaccinated patients (6.89%) for IgG response (Supplementary Fig. 5M) and 3 vaccinated patients (10.34%) for TAb response (Supplementary Fig. 5O) were part of the C3 cluster. Antibody levels (IgG and TAb) were found to be higher at t2 than at t1, and then to drop dramatically at t3 within a month. Once again, the biological relevance was made more problematic by the fact that initially classified unvaccinated patients received their first and second doses of vaccine, and vaccinated patients received their booster shot between the first and second intervals of follow-up. Therefore, these two kidney disease clusters represented the adulterated clusters in this sub-cohort.

The gradual decrease in IgG and TAb levels in patients with diabetes in clusters C2 and C1, as well as patients with kidney disease in both clusters of C2, raised important concerns and demanded further clinical history of these patients, which can provide a plausible explanation for their gradually declining IgG levels. Interestingly, additional follow-up found that, 61.11% diabetic patients in C2 for IgG response (Figs. 5C) and 50% in C1 for TAb response (Fig. 5F) were diagnosed with secondary kidney disease. Similar phenomena was observed for the kidney disease sub-cohort where 69.23% kidney disease patients in C2 for IgG response (Figs. 5I) and 58.33% in C1 for TAb response (Fig. 5L) were diagnosed with secondary diabetes. As the majority of these patients were later diagnosed with a secondary comorbid condition, it further corroborates our hypothesis that multimorbidity with diabetes and kidney disease may contribute to a rapid decline of antibody response in fully vaccinated individuals. In summary, these results highlight the possible contribution of the combined effect of diabetes and kidney disease to rapidly reduce the antibody response in fully vaccinated individuals. The patient-wise Z-scores for each cluster in the diabetic and kidney disease cohort are given in Supplementary Table 7 and Supplementary Table 8. The summary statistic is provided in Supplementary Table 9.

## 4 Discussion

COVID-19 fatalities in low- and middle-income countries (LMICs) heavily contribute to the global disease burden due to poor healthcare infrastructure, a lack of human resources, and proper health surveillance. Many enlisted LMICs lacked the essential supplies to locally manufacture vaccines. These countries essentially aimed to instill high vaccination coverage by purchasing vaccines within an affordable price range through COVAX facility and the African Union [9,27,28]. However, constantly waning immunity in fully vaccinated individuals, and rapid decline of antibodies in immunocompromised calls for effective vaccine roll-out and booster administration. Therefore, priority-based vaccine allocation is considered a means to effectively distribute vaccine doses based on priority in recourse-constrained LMICs. For instance, age-based priority under the assumption that prioritizing the administration of the vaccine for elderly people may minimize COVID-19 associated deaths and hospitalizations [29]. Similar approaches are advocated by WHO [30]. Our study urges that the age-based priority may be augmented by multimorbidity-based priority in LMICs that are short of COVID vaccine doses. The primary objective of this study was to explore the decline of vaccine induced antibody response in patients with comorbidity and multimorbidity. Further, the cumulative impact of prior infections and breakthrough infections on the vaccine response was investigated.

The clinical history of vaccination and natural SARS-CoV-2 infection were used to divide participants into two groups for the purpose of assessing the impact of “hybrid immunity”. Our study documented 1.5-fold higher IgG and 1.2-fold higher TAb titers in response to COVID-19 vaccination in individuals previously infected with SARS-CoV-2 compared with individuals having no history of previous infections. Previously, it has been shown that the pre-existing immunity against the virus has been implicated in the expansion of the durability of vaccine-induced antibody responses 6 months post-vaccination, with the half-life of antibodies in previously infected individuals (t_1/2_ = 95, 89, 88 days) being almost two-fold higher compared to that of their uninfected counterparts (t_1/2_ = 47, 45, 52 days). Additionally, RBD-specific memory B cell response along with robust IgG and IgA titers were reported to be induced by vaccination in higher amount in pre-infected individuals rather than the uninfected control groups [31,32]. In case of breakthrough infection, we have observed 1.8-fold higher IgG and 1.2-fold higher TAb titers in post-infected subjects. A study found that breakthrough infection with the Delta variant had 28-fold higher binding antibody titers and 34-fold higher neutralizing antibody titers than vaccinated individuals who tested negative for SARS-CoV-2 [33]. The strength of the nAb titer induced after two doses of vaccine waned over time, while breakthrough infection boosted the level. A study done using five pseudo-type lentivirus (D614G, α, β, δ, ο) indicates nearly sixfold higher neutralizing titer 50% (NT_50_) value at 6 months post vaccination, compared with subjects with no indication of infection [34]. Where Beta and Delta variants manifested the highest resistance to pre-existing immunity against the virus [35,36], a breakthrough infection with the Delta variant infection has been suggested to act as an effective booster providing broad protection against circulating variants of concern (VOCs), including the Omicron variant [37].

Heterogeneity of antibody responses in SARS‐CoV‐2 vaccination recipients with underlying diseases, such as cancer [38], kidney diseases [39,40], chronic liver disease [41], autoimmune disease [42], and diabetes mellitus [43], can be observed in real‐world studies. Earlier studies demonstrated that individuals with chronic kidney disease had a lower antibody response to the Pfizer-BioNTech vaccine [44], and people with diabetes mellitus also showed a lower antibody response after receiving one or two doses of the COVID-19 vaccine [43]. An observational study done in a cohort of 1645 participants suggests that humoral responses following SARS-CoV-2 vaccination are impaired in patients with certain chronic diseases like cancer, diabetes, and chronic kidney diseases. RBD-IgG seroconversion (IgG antibodies against the receptor-binding domain) was lower in these chronic conditions [45]. Our results validated these findings, as humoral responses in patients with comorbidities gradually decayed within 8 months after the second dose of vaccination. However, in contrast to healthy subjects, patients with diabetes, kidney disease, hypertension, and heart disease had somewhat higher median IgG and TAb levels. Consistent with our findings, a study about the association of comorbid illnesses with an altered adaptive immune response to SARS-CoV-2 revealed that patients with comorbidities were able to initiate a more effective polyfunctional antibody response against S, RBD, and N antigens than those without comorbidities. According to the study, several antibody and T cell features were found to be consistently higher in magnitude and to have increased functional breadth among hospitalized subjects with different comorbidities [46]. Another study regarding the impact of specific comorbidities on the cellular immune response documented a scenario where T, B, and NK cell subsets were significantly elevated in nonalcoholic fatty liver disease, as well as in some particular comorbidity groups such as diabetes, hyperlipidemia, hyperuricemia, and gout [47]. Despite having a higher initial antibody titer than healthy individuals, we have further presented data showing that, patients with kidney disease and diabetes mellitus experience significantly diminished TAb and IgG antibody responses following vaccination. Antibody monitoring of these patients at subsequent intervals showed a gradual decline of antibody titers within 4 months after receiving the second dose of COVID-19 vaccine. Additional investigation into their clinical background uncovered a coexisting chronic secondary condition. Hence, patients with concomitant diabetes and kidney disease exhibit a rapid decline in antibody titers after vaccination, thus rendering these patients with multiple disease conditions vulnerable even after complete vaccination doses.

The relationship between diabetic kidney disease and COVID-19 is not fully resolved. Diabetes mellitus is responsible for changes in innate and adaptive immunity, including aberrant cytokine responses, a reduction in leukocyte recruitment, and neutrophil dysfunction [48]. Moreover, chronic, low-grade inflammation is linked to kidney disease and may make COVID-19 symptoms worse. This inflammation is caused by a number of reasons, including increased levels of the cytokines IL-6 and CRP, oxidative stress, and decreased metabolism [49]. Therefore, the underlying pathogenesis of chronic kidney disease may increase vulnerability to hyperinflammation and cytokine storm upon SARS-CoV-2 infection, resulting in the waning of antibodies in patients with kidney disease, while aberrant immune responses may contribute to the declining nature of antibodies in these comorbid patients with diabetes. Further research is needed to fully understand the mechanisms behind this phenomenon and to identify strategies for improving antibody responses in these vulnerable populations.

### 4.1 Strengths of the study

Previous studies have been done on the variability of antibody response in different disease states such as cancer [38,50], diabetes mellitus [43,51], kidney disease [52], and autoimmune disease [42,53]. Our study simultaneously examined the heterogeneity of antibody response in a variety of chronic diseases, including diabetes, kidney disease, hypertension, cardiovascular disease, autoimmune disease, and cancer. The simultaneous comparison of antibody titer waning in these disease conditions will help establish priority basis for vaccination among these disease states. Additionally, the study also included multimorbid patients having both kidney disease and diabetes to observe the synergistic effect on antibody response decline, which revealed the increased vulnerability of these patients due to increased antibody titer decline. Previously, numerous studies focused on the relation of comorbidities with the outcome of COVID-19 from Bangladesh and other LMIC countries [[54], [55], [56], [57]]. To our knowledge, however, there are no cohort studies on LMIC concentrating on the decline of antibody titers in distinct comorbid and multimorbid populations. Our comprehensive study is the first to elucidate and compare the antibody titer waning among different comorbid and multimorbid groups in an LMIC country. Furthermore, the study used chemiluminescent Siemens Dimension SARS- CoV-2 IgG (CV2G) and SARS-CoV-2 Total Antibody Assays (CV2T) to measure antibody levels. These tests were shown to have high sensitivity and specificity (100% CV2G specificity and sensitivity; 100% and 99.8% CV2T sensitivity and specificity, respectively) as indicated by FDA emergency use authorized (EUA) test performance [18]. In addition, the research comparing three fully automated SARS-CoV-2 antibody assays revealed that the Siemens assay was marginally more sensitive than the other platforms (Roche and Euroimmun assays) [58]. Thus, the use of both Siemens CV2T and CV2G tests with higher accuracy strengthens the validity of the results in this study.

### 4.2 Limitations of the study

There are a number of significant limitations to this study. First, the study did not gather data on other factors that may influence immune response, such as the use of immunosuppressive medications or pharmaceuticals administered for co-morbidities. Statins and metformin have been reported to impair the antibody response and efficacy of vaccines [59,60], and information on these administrations was also lacking. Previous research identified CD4^+^ T cells as the hallmark of a vaccine-induced SARS-CoV-2–specific T cell response [61,62], in particular TNF-α and IL-2 producing CD4^+^ T cells [63]. Therefore, in addition to analyzing humoral responses, an evaluation of T cell responses in these immunosuppressed patients would have been crucial for better understanding the immunological responses to vaccination and previous infections; however, our study design precluded this investigation. At the time of this study, the correlation of neutralization titers using plaque-reduction neutralization tests (PRNT) for CV2G and CV2T kits was still under development [64]. Secondly, the uninfected population in this study represented the non-symptomatic vaccinated patients. As their non-symptomatic status was confirmed only by oral testimony, we ought to have employed more specific diagnostic criteria, such as an anti-SARS-CoV-2 nucleoprotein (N) test. It has been demonstrated that serological responses to nucleoprotein are effective in determining the incidence of SARS-CoV-2 infections in a population that is only minimally symptomatic or does not exhibit any symptoms at all [65,66]. Therefore, this sub-category may represent a mixed population of asymptomatic and true vaccinated individuals. Thirdly, this study was able to follow up on a small number of patients with diabetes and kidney disease. In spite of the fact that these patients agreed to take part in follow-up investigations, they were not controlled because of the ethical significance of this research. This is why the antibody response was stratified using an unsupervised machine learning approach. In certain situations, the date of the second dosage of the vaccine was also lacking. Because so many families had had such a tragic loss, we were unable to reach these patients via phone call. Finally, the study's ability to generalize its findings to a larger population was constrained by a small sample size of patients with co-morbidities. Due to false-positives, some samples had to be eliminated, thus reducing the sample size. The study was carried out at a single facility in Dhaka, Bangladesh; therefore, it might not be representative of other people or environments. The study's exclusive emphasis on Bangladeshi patients may have limited its capacity to generalize to other populations.

## 5 Conclusion

These observations endorsed the outcome of our study, based on which we conclude that kidney disease combined with diabetes serves as a crucial risk factor for the decline of vaccine response in patients. Taking all the evidence into consideration, we propose that, in addition to age-based priority, patients suffering from concomitant diabetes and kidney disease should be prioritized for vaccine and booster dose administration in the LMICs. The findings of this study will assist in shifting the priority basis for vaccination and booster dose administration in comorbid and multimorbid patients.

## Patient and public involvement statement

All the participants were enlisted for the study after obtaining informed written consent. Patients were enrolled at the time of sample collection after examining their medical histories.

## Research ethics approval

The protocols were approved by the institutional ethics committee at Central Police Hospital, Dhaka, and complied with the International Conference on Harmonisation Guideline for Good Clinical Practice and the Declaration of Helsinki. All patients provided written informed consent according to local guidelines. Reference number- CPHERC/R/02/2021.

## Author contribution statement

Depro Das: Conceived and designed the experiments; Performed the experiments; Analyzed and interpreted the data; Contributed reagents, materials, analysis tools or data; Wrote the paper.

Fahmida Khanam Raha: Contributed reagents, materials, analysis tools or data; Wrote the paper.

Khondekar Mustaq Adnan; Md. Rubayet Siraj; Mariam Jamila Shapla; Farzana Shumy; Md. Emdadul Haque; Monwar Hasanat Khan; Susmita Sanyal; Md. Ismail Hosen; AHM Nurun Nabi; Mousumi Sanyal; Md. Zahid Amin: Conceived and designed the experiments; Contributed reagents, materials, analysis tools or data.

Sajib Chakraborty: Conceived and designed the experiments; Analyzed and interpreted the data; Contributed reagents, materials, analysis tools or data; Wrote the paper.

## Data availability statement

Data will be made available on request.

## Declaration of competing interest

The authors declare that they have no known competing financial interests or personal relationships that could have appeared to influence the work reported in this paper.

## Appendix A Supplementary data

Supplementary Figure 1 **IgG and TAb response with respect to gender and age.** Comparison of SARS-CoV-2 specific IgG and TAb response between male and female in the Vaccinated (A, B), Unvaccinated (C, D), and Naturally-infected (E, F) groups. Comparison of IgG and TAb response between adults (<60) and elders (≥60) in all patients (G, H), Vaccinated (I, J), Unvaccinated (K, L), and Naturally-infected (M, N) groups. The box-plots show the 25th to 75th percentiles, the horizontal line represents the median, and the whiskers are calculated by hinge ± 1.5 x IQR. Mann-Whitney U test was used for pairwise comparisons between two gender groups. Spearman Correlation Coefficient test was used to determine the rho (ρ) and P-values in two age groups. The dashed line represents the specimen cutoff value. Supplementary Figure 1

Supplementary Figure 2 **IgG and TAb response among Unvaccinated and Naturally-infected groups with respect to different comorbidities.** SARS-CoV-2 specific IgG (A) and TAb (B) response in the Unvaccinated-group alongside IgG (C) and TAb (D) response in Naturally-infected group, classified by different comorbidities (Diabetes, Hypertension, Heart Disease, Kidney Disease, Cancer, Autoimmune Disease). The box plots show the 25th to 75th percentiles, the horizontal line represents the median, and the whiskers are calculated by hinge ± 1.5 x IQR. P-values were calculated using Kruskal–Wallis H test with Dunn’s posthoc alongside Bonferroni adjustment. The dashed line represents the specimen cutoff value. Supplementary Figure 2

Supplementary Figure 3 **The decline of IgG and TAb response over time in vaccinated individuals.** SARS-CoV-2 specific IgG (A) and (B) TAb response in different time points (0-8 months) after the second dose of vaccine. Each boxplot represents one-time point. Box plots represent 25th to 75th percentiles, the horizontal line represents the median, and the whiskers are calculated by hinge ± 1.5 x IQR. Individual antibody levels are plotted as single dots. P-values were calculated using Kruskal–Wallis H test with Dunn’s posthoc alongside Bonferroni adjustment. The dashed line represents the specimen cutoff value. Supplementary Figure 3

Supplementary Figure 4 **The Decline of IgG and TAb response over time in patients with comorbidities.** Scatter-plot for SARS-CoV-2 specific IgG and TAb response in vaccinated individuals with different comorbidities. IgG response in patients with heart disease (A), and cancer (B). TAb response in patients with hypertension (C), autoimmune disease (D), heart disease (E), and cancer (F). Each dot represents antibody level after second dose of vaccine in an individual patient. P values and rho (ρ) were determined using Spearman Correlation Coefficient test. Only statistically insignificant associations (p>0.05) are shown. The dashed line represents the specimen cutoff value. Supplementary Figure 4

Supplementary Figure 5 **Patient-specific temporal variation of IgG and TAb levels in patients with Diabetes and Kidney Disease in adulterated clusters.** The profile plots represent the adulterated trends of IgG and TAb response in diabetic and kidney disease patients. Adulterated IgG response for C1 (A) and C3 (E), and TAb response for C2 (C) and C3 (G) in diabetic patients. Adulterated IgG response for C1 (I) and C3 (M), and TAb response for C1 (K) and C3 (O) in diabetic patients. The percentage of diabetic patients diagnosed with secondary kidney disease for IgG response in C1 (B) and C3 (F), and TAb response in C2 (D) and C3 (H). The percentage of kidney disease patients diagnosed with secondary diabetes for IgG response in C1 (J) and C3 (N), and TAb response in C1 (L) and C3 (P). Supplementary Figure 5
