# Supplementary figures and images for: Dynamic antibody response in SARS-CoV-2 infected patients and COVID-19 vaccine recipients alongside vaccine effectiveness in comorbid and multimorbid groups
Source: Heliyon. 2023 May 20;9(5):e16349. doi: 10.1016/j.heliyon.2023.e16349 (PMC10199753; doi:10.1016/j.heliyon.2023.e16349)

# Supplementary Figure 1

## Gender Groups

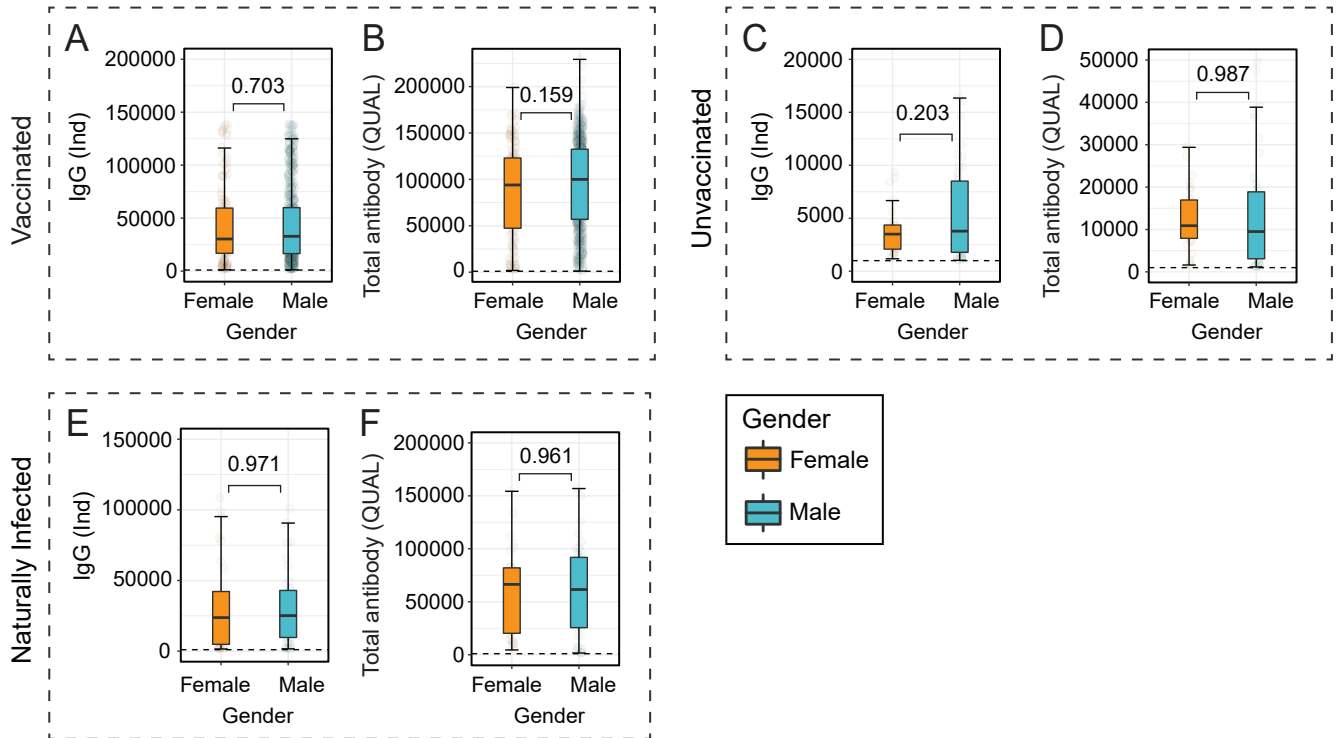

## Age Groups

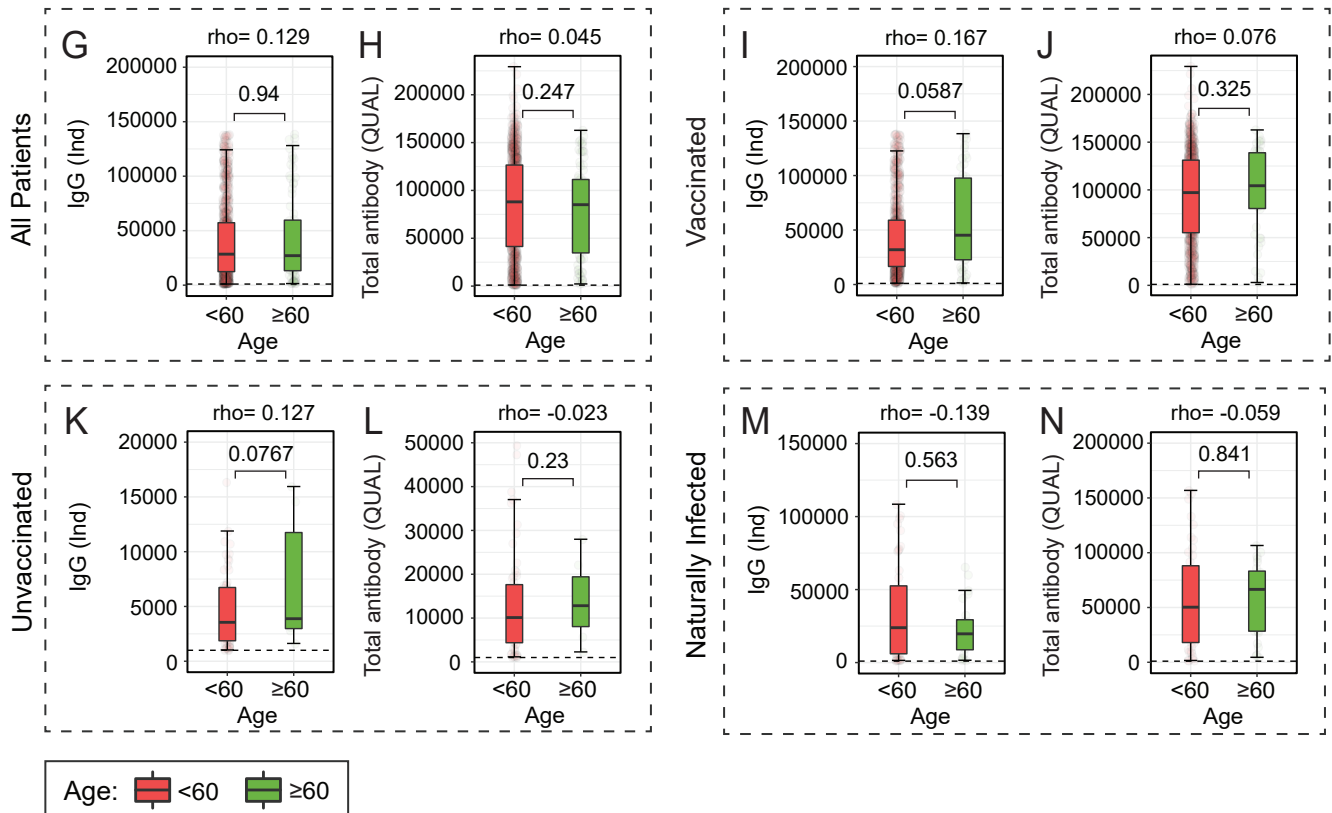

Supplement: Supplementary Figure 1 — IgG and TAb response with respect to gender and age. Comparison of SARS-CoV-2 specific IgG and TAb response between male and female in the Vaccinated (A, B), Unvaccinated (C, D), and Naturally-infected (E, F) groups. Comparison of IgG and TAb response between adults (<60) and elders (≥60) in all patients (G, H), Vaccinated (I, J), Unvaccinated (K, L), and Naturally-infected (M, N) groups. The box-plots show the 25th to 75th percentiles, the horizontal line represents the median, and the whiskers are calculated by hinge ± 1.5 x IQR. Mann-Whitney U test was used for pairwise comparisons between two gender groups. Spearman Correlation Coefficient test was used to determine the rho (ρ) and P-values in two age groups. The dashed line represents the specimen cutoff value. [file mmc1.pdf]

Supplementary Figure 2

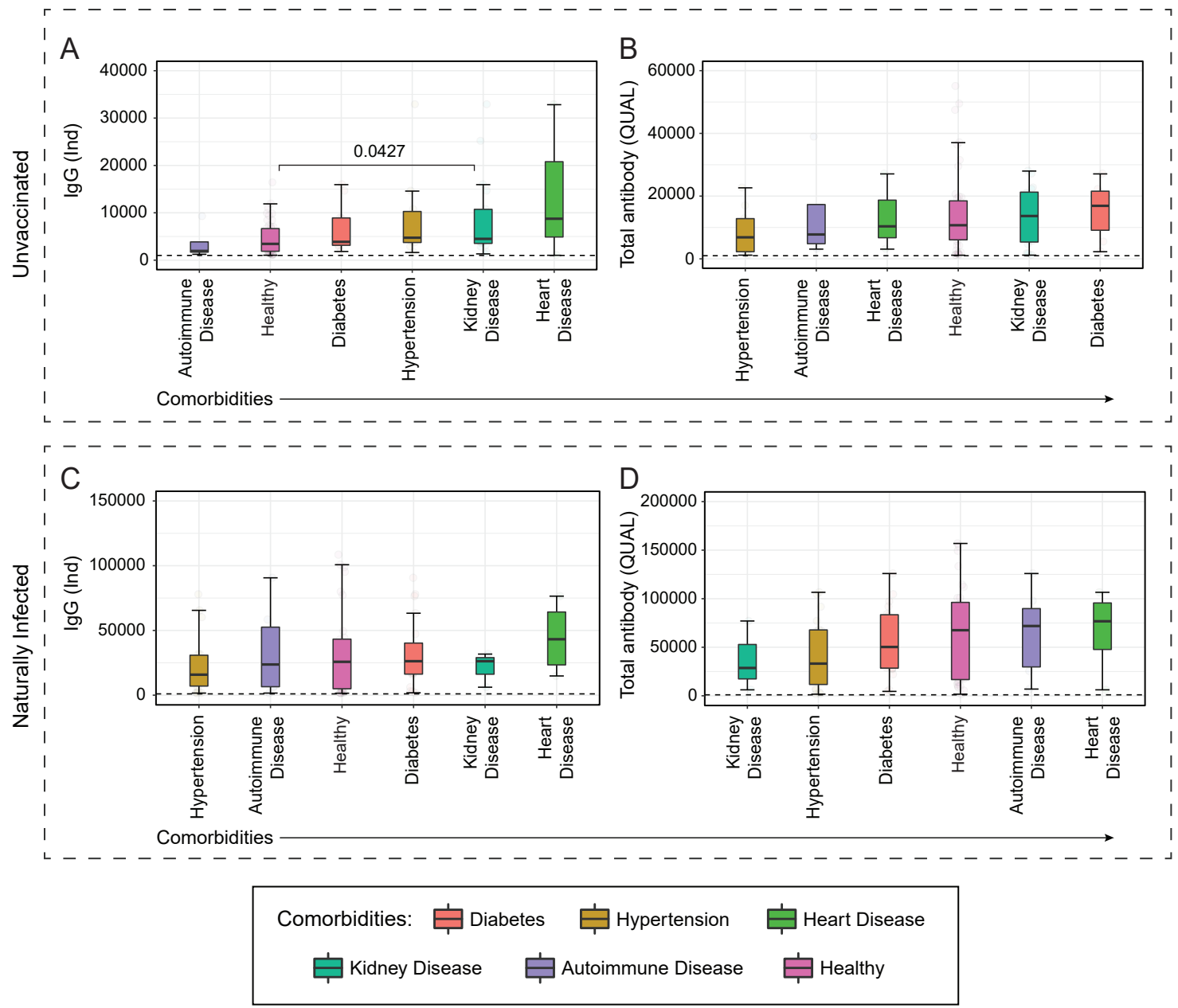

Supplement: Supplementary Figure 2 — IgG and TAb response among Unvaccinated and Naturally-infected groups with respect to different comorbidities. SARS-CoV-2 specific IgG (A) and TAb (B) response in the Unvaccinated-group alongside IgG (C) and TAb (D) response in Naturally-infected group, classified by different comorbidities (Diabetes, Hypertension, Heart Disease, Kidney Disease, Cancer, Autoimmune Disease). The box plots show the 25th to 75th percentiles, the horizontal line represents the median, and the whiskers are calculated by hinge ± 1.5 x IQR. P-values were calculated using Kruskal–Wallis H test with Dunn’s posthoc alongside Bonferroni adjustment. The dashed line represents the specimen cutoff value. [file mmc2.pdf]

Supplementary Figure 3

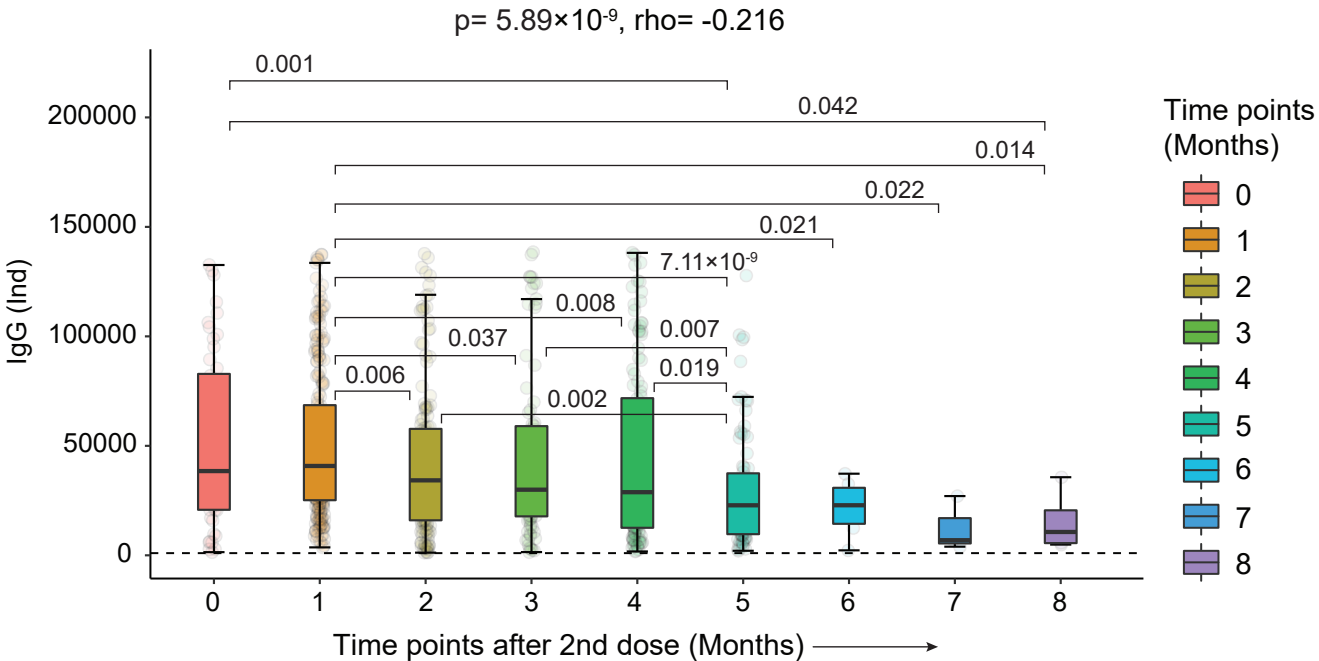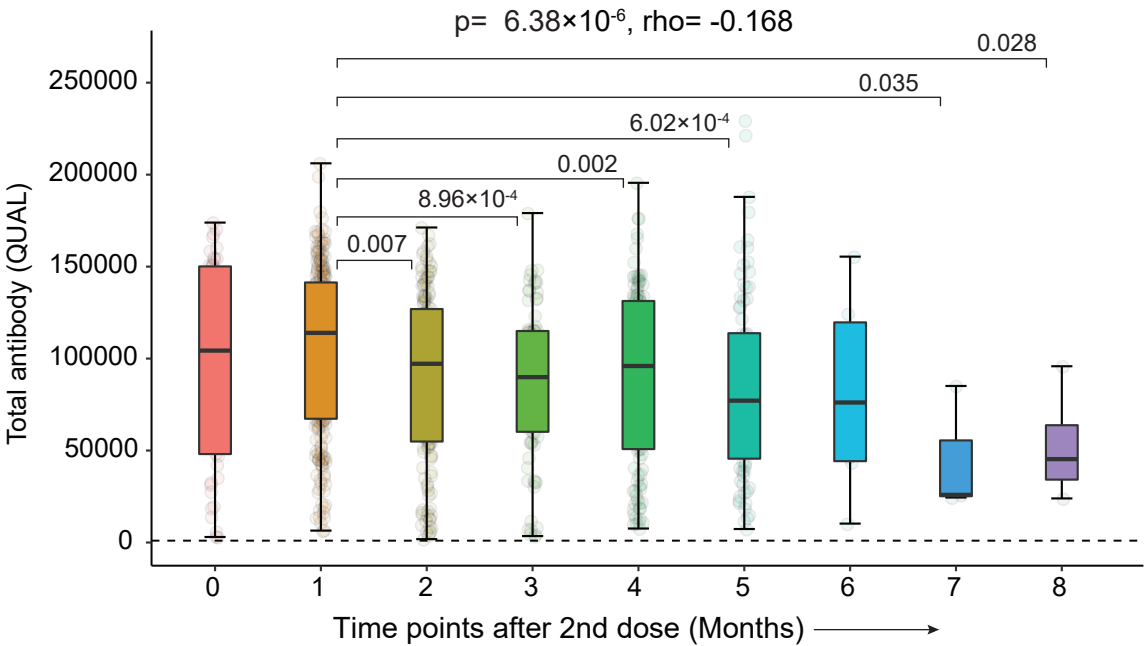

Supplement: Supplementary Figure 3 — The decline of IgG and TAb response over time in vaccinated individuals. SARS-CoV-2 specific IgG (A) and (B) TAb response in different time points (0-8 months) after the second dose of vaccine. Each boxplot represents one-time point. Box plots represent 25th to 75th percentiles, the horizontal line represents the median, and the whiskers are calculated by hinge ± 1.5 x IQR. Individual antibody levels are plotted as single dots. P-values were calculated using Kruskal–Wallis H test with Dunn’s posthoc alongside Bonferroni adjustment. The dashed line represents the specimen cutoff value. [file mmc3.pdf]

Supplementary Figure 4

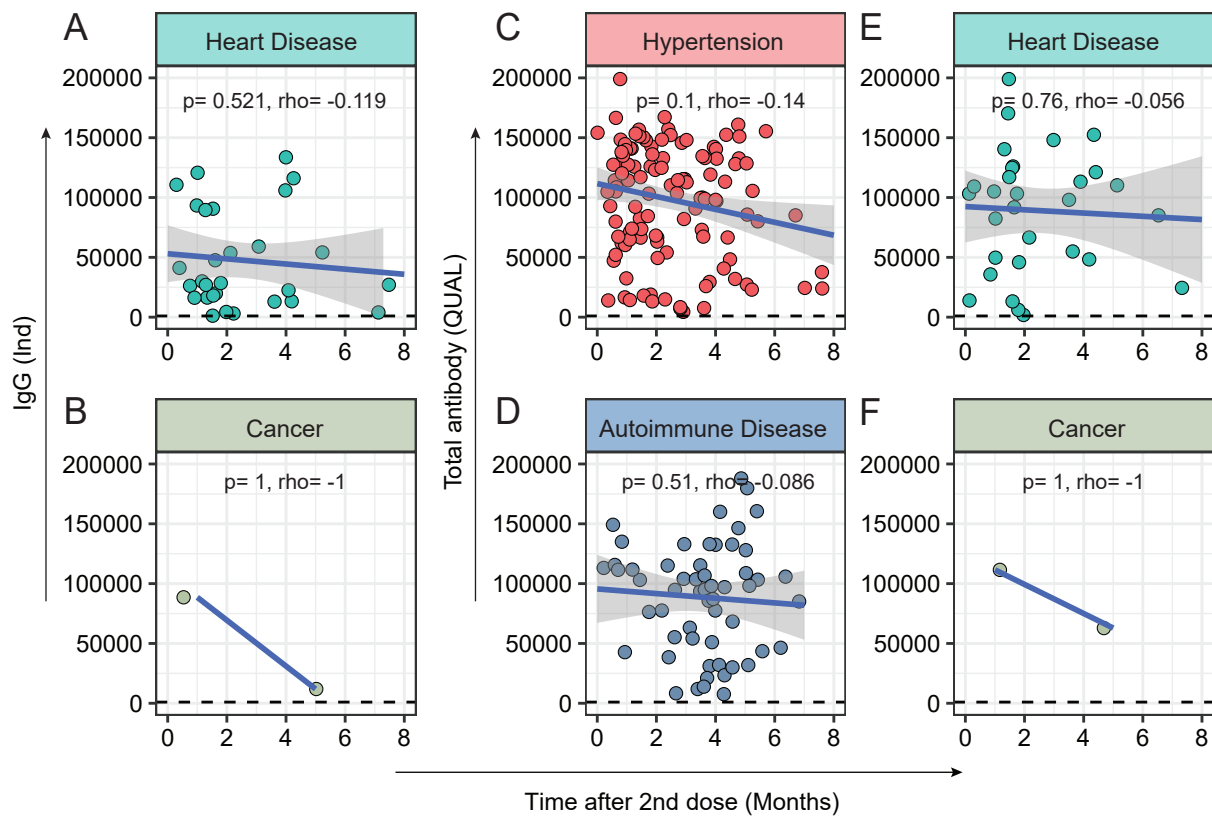

Supplement: Supplementary Figure 4 — The Decline of IgG and TAb response over time in patients with comorbidities. Scatter-plot for SARS-CoV-2 specific IgG and TAb response in vaccinated individuals with different comorbidities. IgG response in patients with heart disease (A), and cancer (B). TAb response in patients with hypertension (C), autoimmune disease (D), heart disease (E), and cancer (F). Each dot represents antibody level after second dose of vaccine in an individual patient. P values and rho (ρ) were determined using Spearman Correlation Coefficient test. Only statistically insignificant associations (p>0.05) are shown. The dashed line represents the specimen cutoff value. [file mmc4.pdf]
