# Supplementary material for: Dynamic antibody response in SARS-CoV-2 infected patients and COVID-19 vaccine recipients alongside vaccine effectiveness in comorbid and multimorbid groups
Source: Heliyon. 2023 May 20;9(5):e16349. doi: 10.1016/j.heliyon.2023.e16349 (PMC10199753; doi:10.1016/j.heliyon.2023.e16349)

Supplementary Figure 5

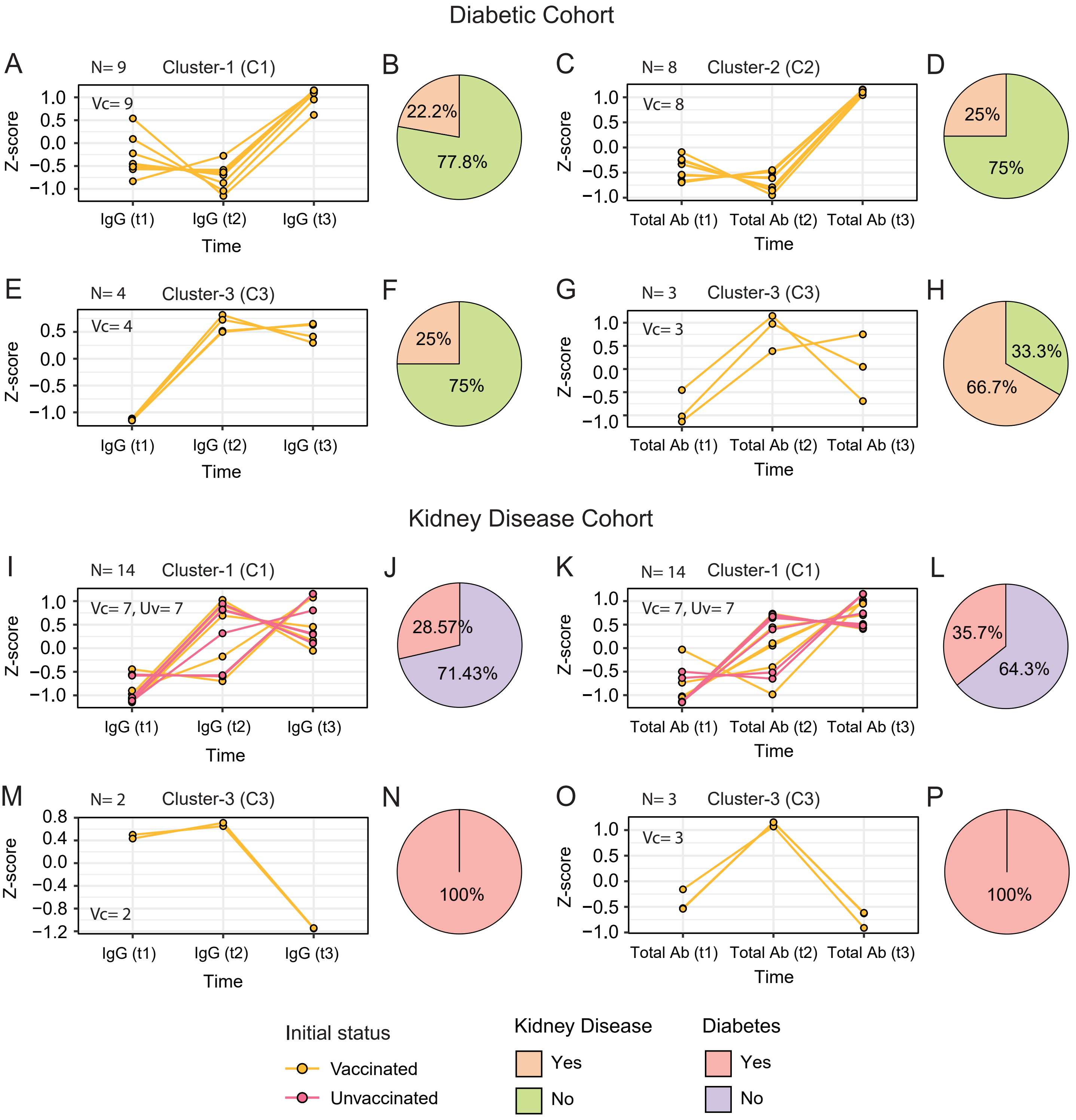

Supplement: Supplementary Figure 5 — Patient-specific temporal variation of IgG and TAb levels in patients with Diabetes and Kidney Disease in adulterated clusters. The profile plots represent the adulterated trends of IgG and TAb response in diabetic and kidney disease patients. Adulterated IgG response for C1 (A) and C3 (E), and TAb response for C2 (C) and C3 (G) in diabetic patients. Adulterated IgG response for C1 (I) and C3 (M), and TAb response for C1 (K) and C3 (O) in diabetic patients. The percentage of diabetic patients diagnosed with secondary kidney disease for IgG response in C1 (B) and C3 (F), and TAb response in C2 (D) and C3 (H). The percentage of kidney disease patients diagnosed with secondary diabetes for IgG response in C1 (J) and C3 (N), and TAb response in C1 (L) and C3 (P). [file mmc5.pdf]
