# Supplementary material for: Dynamic antibody response in SARS-CoV-2 infected patients and COVID-19 vaccine recipients alongside vaccine effectiveness in comorbid and multimorbid groups
Source: Heliyon. 2023 May 20;9(5):e16349. doi: 10.1016/j.heliyon.2023.e16349 (PMC10199753; doi:10.1016/j.heliyon.2023.e16349)
Supplement: Multimedia component 6 [file mmc6.docx]

**Supplementary Tables**

| **Supplementary Table 1. IgG and TAb response in all groups according to gender and age.** | | | | | | | |
| --- | --- | --- | --- | --- | --- | --- | --- |
| All patients (N=912) | | Median Ab | | Interquartile range (IQR) | | 95% Confidence Interval (CI) | |
|  |  | IgG (Ind) | TAb (QUAL) | IgG (Ind) | TAb (QUAL) | IgG (Ind) | TAb (QUAL) |
| Gender  (N=912) | Male (N=719, 78.84%) | 29323 | 91063 | 14033-58393 | 47791-127784 | 38595.5-43734.7 | 84283.77-91460.89 |
|  | Female (N=193, 21.16%) | 25427 | 68191 | 5760-54918 | 19766-114480 | 31078.19-41344.05 | 64720.99-79340.32 |
| Age  (N=906) | ≥60 (N=79, 8.72%) | 27059 | 85102 | 13282-59668 | 34708-111430 | 33949.26-51970.87 | 67696.24-88850.58 |
|  | <60 (N=827, 91.28%) | 28450 | 88051 | 12412-57277 | 41413-85090 | 37505.37-42271.84 | 81660.98-88519.38 |
| Vaccinated (N=771, 84.54%) | |  |  |  |  |  |  |
| Gender  (N=771) | Male (N=637, 82.62%) | 32842 | 100009 | 16518-59894 | 56951-132506 | 41243.73-46733.45 | 90375.75-97585.07 |
|  | Female (N=134, 17.38%) | 30317 | 93969 | 16847-59480 | 47404-123048 | 37364.56-50039.53 | 78758.35-95520.01 |
| Age  (N=766) | ≥60 (N=46, 6.00%) | 45237 | 104355 | 22713-97667 | 80498-138892 | 44454.41-70613.42 | 86101.37-112150.19 |
|  | <60 (N=720, 93.99%) | 31926 | 97149 | 16565-59013 | 55155-131153 | 40586.15-45685.08 | 88993.09-95879.14 |
| Unvaccinated (N=73, 8%) | |  |  |  |  |  |  |
| Gender  (N=73) | Male (N=45, 61.64%) | 4431 | 11283 | 1879-9841 | 4120-31380 | 5366.535-17014.443 | 14165.38-36176.62 |
|  | Female (N=28, 38.36%) | 3521 | 11514 | 2092-4539 | 8208-17552 | 1690.376-9530.267 | 7378.428-26580.786 |
| Age  (N=73) | ≥60 (N=9, 12.33%) | 8915 | 16662 | 3140-15947 | 10361-27987 | -3489.198 – 43876.087 | 3189.865-56801.246 |
|  | <60 (N=64, 87.67%) | 3682 | 10716 | 1881-7063 | 5202-19961 | 4188.050-10778.200 | 12800.77-29016.86 |
| Naturally infected (N=68, 7.46%) | |  |  |  |  |  |  |
| Gender  (N=68) | Male (N=37, 54.41%) | 25126 | 61572 | 9654-42952 | 25589-91931 | 20176.70-37845.03 | 44374.01-73571.12 |
|  | Female (N=31, 45.59%) | 23705 | 66409 | 4810-42192 | 20241-82024 | 19716.69-43224.15 | 41554.08-71338.69 |
| Age  (N=67) | ≥60 (N=24, 35.82%) | 19689 | 66534 | 8778-29329 | 28376-83199 | 15748.28-31380.39 | 42435.06-70386.02 |
|  | <60 (N=43, 64.18%) | 23889 | 50281 | 6020-52543 | 18015-88096 | 23489.66-44013.18 | 43249.33-71979.09 |

| **Supplementary Table 2. IgG and TAb response in all groups according to individual comorbidities.** | | | | | | | |
| --- | --- | --- | --- | --- | --- | --- | --- |
| All patients (N= 912) | | Median Ab | | Interquartile range (IQR) | | 95% Confidence Interval (CI) | |
|  |  | IgG (Ind) | TAb (QUAL) | IgG (Ind) | TAb (QUAL) | IgG (Ind) | TAb (QUAL) |
| Comorbidities | Diabetes (N=152, 16.67%) | 36740 | 99946 | 17040-74699 | 56385-133014 | 42180.83-54177.06 | 86109.33-102056.07 |
|  | Hypertension (N=168, 18.42%) | 37276 | 98054 | 15513-77890 | 36981-127943 | 43649.81-56069.63 | 79211.72-94948.60 |
|  | Heart Disease (N=40, 4.39%) | 32130 | 91931 | 16365-60167 | 45889-113206 | 34337.20-58916.50 | 66691.43-98350.38 |
|  | Kidney Disease (N=73, 8%) | 28364 | 82035 | 9577-59005 | 23962-121172 | 33720.30-51202.17 | 67048.74-92103.12 |
|  | Cancer (N=2, 0.22%) | 50252 | 87190 | 31091-69412 | 75029-99350 | -436669.3–537172.3 | -221844.5–396223.5 |
|  | Autoimmune Disease (N=73, 8%) | 27813 | 93635 | 12427-58390 | 42725-115311 | 30848.57-46817.89 | 74058.37-97504.89 |
|  | Healthy (N=585, 64.14%) | 26823 | 84424 | 10967-52615 | 40932-124837 | 34438.08-39855.99 | 79373.80-87406.25 |
| Vaccinated (N=771) | |  |  |  |  |  |  |
| Comorbidities | Diabetes (N=124, 16.08%) | 45106 | 110201 | 23093-88529 | 72698-138205 | 46867.51-60259.96 | 96049.29-112606.63 |
|  | Hypertension (N=140, 18.16%) | 47555 | 108980 | 23452-90643 | 64899-134482 | 49297.74-62978.80 | 89660.55-105701.92 |
|  | Heart Disease (N=33, 4.28%) | 35671 | 98054 | 18095-89533 | 49720-121172 | 35743.15-64816.97 | 72810.5-107554.9 |
|  | Kidney Disease (N=52, 6.74%) | 50501 | 109567 | 24474-88485 | 75768-136824 | 43384.04-63892.27 | 89830.54-114506.77 |
|  | Cancer (N=2, 0.26%) | 50252 | 87190 | 31091-69412 | 75029-99350 | -436669.3–  537172.3 | -221844.5–396223.5 |
|  | Autoimmune Disease (N=61, 7.91%) | 28393 | 97039 | 12969-58974 | 54137-115622 | 32296.75-49839.77 | 79280.69-103494.03 |
|  | Healthy (N=506, 65.63%) | 28742 | 92385 | 15093-55651 | 53673-129255 | 37363.58-43182.91 | 86386.48-94535.23 |
| Unvaccinated (N=73) | |  |  |  |  |  |  |
| Comorbidities | Diabetes (N=9, 12.33%) | 3869 | 20962 | 3140- 8915 | 12821- 27077 | 3015.144- 11134.856 | 4672.319- 57451.458 |
|  | Hypertension (N=10, 13.69%) | 4738 | 8610 | 3730- 10280 | 3144- 15881 | 2170.974- 15535.826 | 1267.443- 27140.157 |
|  | Heart Disease (N=3, 4.11%) | 8748 | 10361 | 4888- 20788 | 6726- 18719 | -27000.66– 55403.99 | -17041.58–44061.58 |
|  | Kidney Disease (N= 18, 24.66%) | 4738 | 15580 | 3608-13620 | 5649- 22522 | 2391.449-24983.440 | 8227.88-34487.68 |
|  | Autoimmune Disease (N=5, 6.85%) | 2063 | 10132 | 1838-  9224 | 5408-  38854 | -24185.04–62421.04 | -48289.29–145720.09 |
|  | Healthy (N= 47, 64.38%) | 3503 | 11283 | 1880-  6969 | 6602-  19644 | 3460.583-9521.077 | 12396.75-28789.50 |
| Naturally infected (N=68) | |  |  |  |  |  |  |
| Comorbidities | Diabetes (N=19, 27.94%) | 26236 | 50281 | 16260-40240 | 28476-83470 | 19244.85-45201.15 | 39483.49-73579.78 |
|  | Hypertension (N=18, 26.47%) | 15830 | 33152 | 7032-30856 | 11661-67847 | 12076.58-34841.53 | 26783.33-62267.23 |
|  | Heart Disease (N=4, 5.88%) | 32130 | 91931 | 26236-60167 | 61572-100559 | 10299.62-73641.98 | 21991.68-124727.52 |
|  | Kidney Disease (N=3, 4.41%) | 26236 | 28645 | 16192-28982 | 17395-52845 | -12081.15–54822.48 | -52722.02–127277.35 |
|  | Autoimmune Disease (N=7, 10.29%) | 23705 | 71876 | 6522-52543 | 29743-89817 | 2699.017-64178.698 | 23811.13-103004.01 |
|  | Healthy (N= 32, 47.05%) | 25761 | 67546 | 4961-43285 | 16720-96188 | 20285.69- 44413.06 | 45592.41-80270.78 |

**Supplementary Table 3. Pairwise comparisons between different comorbid patient groups for IgG and TAb response for all four groups.**

| All patients | | | | | | |
| --- | --- | --- | --- | --- | --- | --- |
| Comparison groups | IgG (Ind) | | | Tab (QUAL) | | |
|  | Z-value | P-value | P-adj | Z-value | P-value | P-adj |
| Cancer - Diabetes | -0.0615 | 0.9509 | 1 | -0.2383 | 0.8117 | 1 |
| Cancer - Heart Disease | 0.0127 | 0.9897 | 1 | 0.1070 | 0.9148 | 1 |
| Cancer - Hypertension | -0.0586 | 0.9532 | 1 | -0.0615 | 0.9509 | 1 |
| Cancer - Kidney Disease | 0.2122 | 0.8319 | 1 | 0.1359 | 0.8919 | 1 |
| Cancer - Healthy | 0.3555 | 0.7221 | 1 | 0.0275 | 0.9781 | 1 |
| Cancer - Autoimmune Disease | 0.2868 | 0.7742 | 1 | 0.0051 | 0.9960 | 1 |
| Diabetes - Heart Disease | 0.2987 | 0.7651 | 1 | 1.3906 | 0.1643 | 1 |
| Diabetes - Hypertension | 0.0188 | 0.9849 | 1 | 1.1241 | 0.2610 | 1 |
| Diabetes - Kidney Disease | 1.3757 | 0.1688 | 1 | 1.8752 | 0.0608 | 1 |
| Diabetes - Healthy | 3.2476 | 0.0011 | 0.0244 | 2.0767 | 0.0378 | 0.7943 |
| Diabetes - Autoimmune Disease | 1.7513 | 0.0798 | 1 | 1.2164 | 0.2238 | 1 |
| Heart Disease - Hypertension | -0.2897 | 0.7720 | 1 | -0.6894 | 0.4906 | 1 |
| Heart Disease - Kidney Disease | 0.7260 | 0.4678 | 1 | 0.1012 | 0.9194 | 1 |
| Heart Disease - Healthy | 1.4842 | 0.1377 | 1 | -0.3552 | 0.7224 | 1 |
| Heart Disease - Autoimmune Disease | 0.9979 | 0.3183 | 1 | -0.3757 | 0.7072 | 1 |
| Hypertension - Kidney Disease | 1.3825 | 0.1668 | 1 | 1.0072 | 0.3138 | 1 |
| Hypertension - Healthy | 3.3537 | 0.0007 | 0.0167 | 0.7224 | 0.4700 | 1 |
| Hypertension - Autoimmune Disease | 1.7640 | 0.0777 | 1 | 0.3381 | 0.7353 | 1 |
| Kidney Disease - Healthy | 0.8036 | 0.4216 | 1 | -0.6280 | 0.5300 | 1 |
| Kidney Disease - Autoimmune Disease | 0.3231 | 0.7465 | 1 | -0.5667 | 0.5709 | 1 |
| Healthy - Autoimmune Disease | -0.3727 | 0.7093 | 1 | -0.1277 | 0.8984 | 1 |
| Vaccinated | | | | | | |
| Cancer - Diabetes | -0.3240 | 0.7459 | 1 | -0.6119 | 0.5406 | 1 |
| Cancer - Heart Disease | -0.1278 | 0.8983 | 1 | -0.1811 | 0.8563 | 1 |
| Cancer - Hypertension | -0.3625 | 0.7170 | 1 | -0.4431 | 0.6577 | 1 |
| Cancer - Kidney Disease | -0.3426 | 0.7319 | 1 | -0.5630 | 0.5734 | 1 |
| Cancer - Healthy | 0.1753 | 0.8608 | 1 | -0.2362 | 0.8133 | 1 |
| Cancer - Autoimmune Disease | 0.1502 | 0.8806 | 1 | -0.2027 | 0.8394 | 1 |
| Diabetes - Heart Disease | 0.7041 | 0.4814 | 1 | 1.5534 | 0.1203 | 1 |
| Diabetes - Hypertension | -0.2201 | 0.8258 | 1 | 0.9780 | 0.3281 | 1 |
| Diabetes - Kidney Disease | -0.0961 | 0.9234 | 1 | 0.1843 | 0.8538 | 1 |
| Diabetes - Healthy | 3.5446 | 0.0004 | 0.0083 | 2.6827 | 0.0073 | 0.1534 |
| Diabetes - Autoimmune Disease | 2.1669 | 0.0302 | 0.6351 | 1.8575 | 0.0632 | 1 |
| Heart Disease - Hypertension | -0.8530 | 0.3937 | 1 | -0.9492 | 0.3425 | 1 |
| Heart Disease - Kidney Disease | -0.6910 | 0.4896 | 1 | -1.2304 | 0.2186 | 1 |
| Heart Disease - Healthy | 1.2093 | 0.2265 | 1 | -0.1974 | 0.8435 | 1 |
| Heart Disease - Autoimmune Disease | 0.9300 | 0.3524 | 1 | -0.0638 | 0.9491 | 1 |
| Hypertension - Kidney Disease | 0.0694 | 0.9447 | 1 | -0.5551 | 0.5788 | 1 |
| Hypertension - Healthy | 4.0037 | 0.0001 | 0.0013 | 1.5521 | 0.1206 | 1 |
| Hypertension - Autoimmune Disease | 2.3858 | 0.0170 | 0.3579 | 1.1074 | 0.2681 | 1 |
| Kidney Disease - Healthy | 2.5480 | 0.0108 | 0.2275 | 1.6368 | 0.1017 | 1 |
| Kidney Disease - Autoimmune Disease | 1.8796 | 0.0602 | 1 | 1.3777 | 0.1683 | 1 |
| Healthy - Autoimmune Disease | -0.1203 | 0.9042 | 1 | 0.1599 | 0.8729 | 1 |
| Unvaccinated | | | | | | |
| Diabetes - Heart Disease | 0.0218 | 0.9826 | 1 | 0.8396 | 0.4011 | 1 |
| Diabetes - Hypertension | -0.2359 | 0.8135 | 1 | 1.6462 | 0.0997 | 1 |
| Diabetes - Kidney Disease | -0.3084 | 0.7578 | 1 | 0.8792 | 0.3793 | 1 |
| Diabetes - Healthy | 1.1972 | 0.2312 | 1 | 1.2974 | 0.1945 | 1 |
| Diabetes - Autoimmune Disease | 0.7088 | 0.4784 | 1 | 0.5536 | 0.5798 | 1 |
| Heart Disease - Hypertension | -0.1868 | 0.8518 | 1 | 0.2987 | 0.7652 | 1 |
| Heart Disease - Kidney Disease | -0.2252 | 0.8218 | 1 | -0.3220 | 0.7475 | 1 |
| Heart Disease - Healthy | 0.7070 | 0.4795 | 1 | -0.1472 | 0.8830 | 1 |
| Heart Disease - Autoimmune Disease | 0.5214 | 0.6021 | 1 | -0.3436 | 0.7311 | 1 |
| Hypertension - Kidney Disease | -0.0443 | 0.9647 | 1 | -1.0077 | 0.3136 | 1 |
| Hypertension - Healthy | 1.5621 | 0.1183 | 1 | -0.8164 | 0.4143 | 1 |
| Hypertension - Autoimmune Disease | 0.9197 | 0.3577 | 1 | -0.8172 | 0.4138 | 1 |
| Kidney Disease - Healthy | 2.0257 | 0.0428 | 0.6420 | 0.4082 | 0.6832 | 1 |
| Kidney Disease - Autoimmune Disease | 1.0311 | 0.3025 | 1 | -0.0992 | 0.9210 | 1 |
| Healthy - Autoimmune Disease | -0.0856 | 0.9318 | 1 | -0.3471 | 0.7285 | 1 |
| Naturally infected | | | | | | |
| Diabetes - Heart Disease | -0.6644 | 0.5064 | 1 | -0.3250 | 0.7452 | 1 |
| Diabetes - Hypertension | 1.1121 | 0.2661 | 1 | 1.0350 | 0.3007 | 1 |
| Diabetes - Kidney Disease | 0.4107 | 0.6813 | 1 | 0.7892 | 0.4300 | 1 |
| Diabetes - Healthy | 0.5064 | 0.6126 | 1 | -0.1383 | 0.8900 | 1 |
| Diabetes - Autoimmune Disease | 0.2710 | 0.7864 | 1 | -0.3457 | 0.7295 | 1 |
| Heart Disease - Hypertension | 1.3230 | 0.1858 | 1 | 0.9393 | 0.3476 | 1 |
| Heart Disease - Kidney Disease | 0.8126 | 0.4164 | 1 | 0.8760 | 0.3810 | 1 |
| Heart Disease - Healthy | 0.9658 | 0.3342 | 1 | 0.2616 | 0.7936 | 1 |
| Heart Disease - Autoimmune Disease | 0.7743 | 0.4388 | 1 | 0.0414 | 0.9670 | 1 |
| Hypertension - Kidney Disease | -0.1774 | 0.8592 | 1 | 0.2403 | 0.8101 | 1 |
| Hypertension - Healthy | -0.7438 | 0.4570 | 1 | -1.2914 | 0.1965 | 1 |
| Hypertension - Autoimmune Disease | -0.5523 | 0.5808 | 1 | -1.1075 | 0.2681 | 1 |
| Kidney Disease - Healthy | -0.1797 | 0.8574 | 1 | -0.8783 | 0.3798 | 1 |
| Kidney Disease - Autoimmune Disease | -0.1961 | 0.8445 | 1 | -0.9320 | 0.3513 | 1 |
| Healthy - Autoimmune Disease | -0.0644 | 0.9487 | 1 | -0.2704 | 0.7869 | 1 |

| **Supplementary Table 4. IgG and TAb response in the vaccinated-group over definite time points (months).** | | | | | | | |
| --- | --- | --- | --- | --- | --- | --- | --- |
| Months | Population (N=711) | Median Ab | | Interquartile range (IQR) | | 95% Confidence Interval (CI) | |
|  |  | IgG (Ind) | TAb (QUAL) | IgG (Ind) | TAb (QUAL) | IgG (Ind) | TAb (QUAL) |
| 0 | N=48, 6.75% | 38469 | 104344 | 20786-82860 | 48081-150106 | 40563.85-63364.69 | 81317.38-112170.29 |
| 1 | N=221, 31.08% | 40811 | 113963 | 25162-68579 | 67262-141376 | 46029.49-55005.25 | 98774.25-110638.64 |
| 2 | N=150, 21.09% | 34243 | 97159 | 15994-57751 | 54892-126922 | 36583.05-47680.29 | 83645.36-98467.00 |
| 3 | N=81, 11.39% | 29967 | 89903 | 17787-59005 | 60173-114982 | 36131.78-53279.38 | 77658.77-95571.09 |
| 4 | N=116, 16.31% | 28825 | 95962 | 12568-71773 | 50788-131304 | 37341.76-51887.55 | 79718.27-97199.82 |
| 5 | N=82, 11.53% | 22845 | 77115 | 9654- 37453 | 45566-113798 | 23275.43-34955.23 | 74852.92-96808.83 |
| 6 | N=6, 0.84% | 22862 | 76113 | 14386-30840 | 44231-119679 | 8105.92-35335.413 | 22271.22-139635.45 |
| 7 | N=3, 0.42% | 6901 | 25893 | 5426- 16980 | 25176-55498 | -18606.23 –43879.56 | -40814.04 –131116.71 |
| 8 | N=4, 0.56% | 10656 | 45332 | 5573- 20553 | 34179-63768 | -7283.81 –38223.31 | 3014.14 – 102215.85 |

**Supplementary Table 5. Pairwise comparisons between definite time points (months) for IgG and TAb response in the vaccinated-group.**

| Comparison groups | | IgG (Ind) | | TAb (QUAL) | |
| --- | --- | --- | --- | --- | --- |
| Group1 (Month) | Group2 (Month) | P-value | P-adj | P-value | P-adj |
| Zero | One | 0.823 | 1 | 0.451 | 1 |
| Zero | Two | 0.171 | 1 | 0.388 | 1 |
| Zero | Three | 0.296 | 1 | 0.163 | 1 |
| Zero | Four | 0.191 | 1 | 0.27 | 1 |
| Zero | Five | 0.001 | 0.037 | 0.164 | 1 |
| Zero | Six | 0.08 | 1 | 0.582 | 1 |
| Zero | Seven | 0.075 | 1 | 0.1 | 1 |
| Zero | Eight | 0.042 | 1 | 0.13 | 1 |
| One | Two | 0.006 | 0.2 | 0.007 | 0.219 |
| One | Three | 0.037 | 0.952 | 0.000896 | 0.031 |
| One | Four | 0.008 | 0.239 | 0.002 | 0.074 |
| One | Five | 7.11×10^-09^ | 2.56×10^-07^ | 0.000602 | 0.022 |
| One | Six | 0.021 | 0.596 | 0.248 | 1 |
| One | Seven | 0.022 | 0.596 | 0.035 | 1 |
| One | Eight | 0.014 | 0.426 | 0.028 | 0.88 |
| Two | Three | 0.907 | 1 | 0.297 | 1 |
| Two | Four | 0.819 | 1 | 0.621 | 1 |
| Two | Five | 0.002 | 0.081 | 0.218 | 1 |
| Two | Six | 0.147 | 1 | 0.609 | 1 |
| Two | Seven | 0.07 | 1 | 0.107 | 1 |
| Two | Eight | 0.079 | 1 | 0.094 | 1 |
| Three | Four | 0.586 | 1 | 0.719 | 1 |
| Three | Five | 0.007 | 0.215 | 0.497 | 1 |
| Three | Six | 0.194 | 1 | 0.874 | 1 |
| Three | Seven | 0.078 | 1 | 0.087 | 1 |
| Three | Eight | 0.076 | 1 | 0.087 | 1 |
| Four | Five | 0.019 | 0.551 | 0.55 | 1 |
| Four | Six | 0.311 | 1 | 0.692 | 1 |
| Four | Seven | 0.085 | 1 | 0.125 | 1 |
| Four | Eight | 0.097 | 1 | 0.13 | 1 |
| Five | Six | 0.875 | 1 | 0.875 | 1 |
| Five | Seven | 0.171 | 1 | 0.137 | 1 |
| Five | Eight | 0.255 | 1 | 0.148 | 1 |
| Six | Seven | 0.548 | 1 | 0.381 | 1 |
| Six | Eight | 0.61 | 1 | 0.476 | 1 |
| Seven | Eight | 0.857 | 1 | 0.857 | 1 |

**Supplementary Table 6. IgG and TAb response and pairwise comparisons between three vaccinated sub-groups.**

| Groups | IgG (Ind) | | | Comparison group | P-value |
| --- | --- | --- | --- | --- | --- |
|  | Median Ab | Interquartile range (IQR) | 95% Confidence Interval (CI) |  |  |
| Uninfected | 27813 | 14232-51899 | 34215.07-40305.30 | Postinfected | 1.99×10^-4^ |
| Preinfected | 40703 | 24731-83788 | 47433.03-60612.85 | Uninfected | 3.12×10^-6^ |
| Postinfected | 51527 | 24814-93928 | 47918.59-72075.37 | Preinfected | 0.5711 |
|  | TAb (QUAL) | | |  |  |
| Uninfected | 91063 | 49479-127235 | 83778.94-93322.09 | Postinfected | 0.2010 |
| Preinfected | 109819 | 71745-135179 | 96472.37-111531.17 | Uninfected | 1.82×10^-3^ |
| Postinfected | 109787 | 74716-126462 | 85872.09-108851.17 | Preinfected | 0.4744 |

**Supplementary Table 7. Hierarchical cluster analysis and calculated Z-scores for IgG and TAb in patients with Diabetes.**

| Patients ID | IgG Z-score | | | | TAb Z-score | | | |
| --- | --- | --- | --- | --- | --- | --- | --- | --- |
|  | Cluster | t1 | t2 | t3 | Cluster | t1 | t2 | t3 |
| 140 | Cluster-1 | 0.538398 | -1.15384 | 0.615445 | Cluster-1 | 1.153851 | -0.53857 | -0.61528 |
| 275 | Cluster-2 | 1.154687 | -0.58224 | -0.57245 | Cluster-1 | 1.141059 | -0.72379 | -0.41727 |
| 281 | Cluster-1 | -0.46294 | -0.68465 | 1.147584 | Cluster-1 | 1.153806 | -0.61626 | -0.53754 |
| 297 | Cluster-2 | 1.154665 | -0.58523 | -0.56944 | Cluster-1 | 1.137339 | -0.74143 | -0.39591 |
| 300 | Cluster-2 | 1.14919 | -0.47701 | -0.67218 | Cluster-1 | 0.838093 | 0.268849 | -1.10694 |
| 324 | Cluster-2 | 1.153655 | -0.53428 | -0.61937 | Cluster-1 | 1.14128 | -0.72266 | -0.41862 |
| 376 | Cluster-2 | 1.140342 | -0.41296 | -0.72738 | Cluster-1 | 0.998736 | 0.002523 | -1.00126 |
| 470 | Cluster-3 | -1.15287 | 0.520083 | 0.632783 | Cluster-2 | -0.66554 | -0.48442 | 1.149956 |
| 482 | Cluster-1 | -0.4503 | -0.69568 | 1.145977 | Cluster-2 | -0.3331 | -0.79094 | 1.124037 |
| 550 | Cluster-2 | 1.154648 | -0.56781 | -0.58684 | Cluster-1 | 1.120269 | -0.80251 | -0.31776 |
| 620 | Cluster-2 | 1.154484 | -0.55789 | -0.59659 | Cluster-1 | 1.153629 | -0.61989 | -0.53374 |
| 764 | Cluster-2 | 1.154044 | -0.61073 | -0.54331 | Cluster-1 | 1.137763 | -0.73953 | -0.39823 |
| 853 | Cluster-1 | -0.51838 | -0.63438 | 1.152757 | Cluster-2 | -0.55753 | -0.59695 | 1.154476 |
| 860 | Cluster-2 | 1.147383 | -0.46129 | -0.68609 | Cluster-1 | 1.154218 | -0.5482 | -0.60602 |
| 914 | Cluster-1 | -0.56977 | -0.58489 | 1.154668 | Cluster-2 | -0.54174 | -0.61225 | 1.153983 |
| 935 | Cluster-1 | -0.83237 | -0.2769 | 1.109271 | Cluster-3 | -1.02301 | 0.975279 | 0.047733 |
| 974 | Cluster-2 | 1.154254 | -0.60494 | -0.54932 | Cluster-1 | 0.92736 | -1.0595 | 0.13214 |
| 976 | Cluster-1 | -0.22373 | -0.86919 | 1.092914 | Cluster-2 | -0.25768 | -0.84594 | 1.103623 |
| 984 | Cluster-2 | 1.149732 | -0.66753 | -0.4822 | Cluster-1 | 1.154699 | -0.57922 | -0.57548 |
| 992 | Cluster-3 | -1.11471 | 0.818241 | 0.296472 | Cluster-1 | 1.124016 | -0.79101 | -0.33301 |
| 1015 | Cluster-3 | -1.14048 | 0.726676 | 0.413808 | Cluster-3 | -1.13564 | 0.386856 | 0.74878 |
| 1091 | Cluster-2 | 1.152409 | -0.63917 | -0.51324 | Cluster-1 | 1.154 | -0.61182 | -0.54218 |
| 1105 | Cluster-1 | -0.5164 | -0.63623 | 1.152626 | Cluster-2 | -0.69635 | -0.44953 | 1.145874 |
| 1106 | Cluster-2 | 1.121435 | -0.79902 | -0.32242 | Cluster-1 | 1.127662 | -0.3487 | -0.77897 |
| 1118 | Cluster-2 | 1.116126 | -0.81438 | -0.30175 | Cluster-2 | -0.09211 | -0.95076 | 1.042868 |
| 1122 | Cluster-2 | 1.049503 | -0.10773 | -0.94177 | Cluster-1 | 1.148657 | -0.67651 | -0.47215 |
| 1136 | Cluster-1 | 0.090485 | -1.04217 | 0.951682 | Cluster-1 | 1.097912 | -0.8587 | -0.23921 |
| 1138 | Cluster-3 | -1.15133 | 0.499367 | 0.651967 | Cluster-2 | -0.24012 | -0.85808 | 1.098198 |
| 1158 | Cluster-2 | 1.147092 | -0.45894 | -0.68815 | Cluster-1 | 1.13578 | -0.38761 | -0.74817 |
| 1176 | Cluster-2 | 0.734286 | 0.40462 | -1.13891 | Cluster-1 | 1.140644 | -0.41477 | -0.72588 |
| 1178 | Cluster-2 | 0.318657 | 0.801839 | -1.1205 | Cluster-3 | -0.45419 | 1.146488 | -0.6923 |

**Supplementary Table 8. Hierarchical cluster analysis and calculated Z-scores for IgG and TAb in patients with Kidney Disease.**

| Patients ID | IgG Z-score | | | | TAb Z-score | | | |
| --- | --- | --- | --- | --- | --- | --- | --- | --- |
|  | Cluster | t1 | t2 | t3 | Cluster | t1 | t2 | t3 |
| 219 | Cluster-1 | -1.11848 | 0.807728 | 0.310756 | Cluster-1 | -1.14466 | 0.7039 | 0.440763 |
| 466 | Cluster-2 | 1.154662 | -0.56913 | -0.58553 | Cluster-2 | 1.144764 | -0.44148 | -0.70329 |
| 474 | Cluster-1 | -0.44625 | -0.69918 | 1.145429 | Cluster-1 | -0.03081 | -0.98424 | 1.015049 |
| 477 | Cluster-2 | 1.154693 | -0.57366 | -0.58103 | Cluster-2 | 1.144674 | -0.44084 | -0.70383 |
| 568 | Cluster-2 | 1.126741 | -0.34464 | -0.7821 | Cluster-2 | 1.121573 | -0.32297 | -0.7986 |
| 647 | Cluster-1 | -1.12008 | 0.316996 | 0.803081 | Cluster-1 | -1.13792 | 0.399097 | 0.738823 |
| 657 | Cluster-1 | -1.05085 | 0.939894 | 0.110957 | Cluster-1 | -1.14298 | 0.713616 | 0.429363 |
| 663 | Cluster-1 | -1.04806 | 0.943766 | 0.104293 | Cluster-1 | -1.15224 | 0.641364 | 0.510877 |
| 669 | Cluster-2 | 1.119176 | -0.31345 | -0.80573 | Cluster-2 | 1.120649 | -0.31926 | -0.80138 |
| 670 | Cluster-1 | -1.14633 | 0.69334 | 0.452992 | Cluster-1 | -1.14479 | 0.441638 | 0.703149 |
| 673 | Cluster-2 | 1.152354 | -0.51246 | -0.63989 | Cluster-2 | 1.144109 | -0.43692 | -0.70719 |
| 674 | Cluster-3 | 0.499554 | 0.651796 | -1.15135 | Cluster-2 | 0.785515 | 0.340199 | -1.12571 |
| 678 | Cluster-1 | -1.06093 | 0.925201 | 0.135732 | Cluster-1 | -1.13961 | 0.730951 | 0.408659 |
| 915 | Cluster-1 | -0.97236 | 1.025522 | -0.05316 | Cluster-2 | 1.113346 | -0.29145 | -0.8219 |
| 944 | Cluster-2 | 1.147357 | -0.46107 | -0.68628 | Cluster-3 | -0.52534 | 1.153183 | -0.62784 |
| 1004 | Cluster-2 | 1.149097 | -0.47615 | -0.67294 | Cluster-2 | 0.919528 | 0.145092 | -1.06462 |
| 1006 | Cluster-2 | 1.124549 | -0.33525 | -0.7893 | Cluster-1 | -1.02598 | 0.054155 | 0.971822 |
| 1010 | Cluster-2 | 1.140971 | -0.41674 | -0.72424 | Cluster-3 | -0.1584 | 1.069746 | -0.91135 |
| 1013 | Cluster-1 | -0.55892 | -0.59559 | 1.154506 | Cluster-1 | -0.50103 | -0.65044 | 1.151474 |
| 1016 | Cluster-1 | -0.56315 | -0.59143 | 1.154585 | Cluster-1 | -0.73426 | -0.40465 | 1.138912 |
| 1017 | Cluster-1 | -1.11385 | 0.82055 | 0.293304 | Cluster-1 | -1.14951 | 0.669448 | 0.480064 |
| 1018 | Cluster-2 | 1.126183 | -0.34222 | -0.78396 | Cluster-2 | 0.887721 | 0.195642 | -1.08336 |
| 1019 | Cluster-2 | 1.12408 | -0.33328 | -0.7908 | Cluster-2 | 0.786706 | 0.338645 | -1.12535 |
| 1020 | Cluster-1 | -0.5806 | -0.5741 | 1.154694 | Cluster-1 | -0.63534 | -0.51735 | 1.152689 |
| 1021 | Cluster-1 | -0.90024 | -0.17612 | 1.076361 | Cluster-1 | -1.04762 | 0.103251 | 0.944369 |
| 1022 | Cluster-1 | -1.07158 | 0.908328 | 0.163254 | Cluster-2 | 0.711912 | 0.431373 | -1.14328 |
| 1023 | Cluster-2 | 0.925823 | -1.06052 | 0.1347 | Cluster-3 | -0.53772 | 1.153814 | -0.61609 |
| 1024 | Cluster-3 | 0.432956 | 0.710567 | -1.14352 | Cluster-1 | -1.15036 | 0.661831 | 0.488526 |
| 1026 | Cluster-2 | 1.146617 | -0.45519 | -0.69143 | Cluster-2 | 1.120159 | -0.31732 | -0.80284 |

| **Supplementary Table 9. Patient-specific temporal variation of IgG and TAb levels in patients with Diabetes and Kidney Disease.** | | | | | | | |
| --- | --- | --- | --- | --- | --- | --- | --- |
| Statistical Parameters | | Diabetic patients (N=31) | | | Kidney Disease patients (N= 29) | | |
|  |  | Cluster-1 (C1) | Cluster-2 (C2) | Cluster-3 (C3) | Cluster-1 (C1) | Cluster-2 (C2) | Cluster-3 (C3) |
| Median Ab (t1) | IgG (Ind) | -0.4629 | 1.1495 | -1.146 | -1.0495 | 1.1410 | 0.4663 |
|  | TAb (QUAL) | 1.1392 | -0.4374 | -1.0230 | -1.1387 | 1.1168 | -0.5253 |
| Interquartile range (IQR) (t1) | IgG (Ind) | -0.5184 – -0.2237 | 1.1262 – 1.1542 | -1.152 – -1.134 | -1.1033 – -0.6605 | 1.1245 – 1.1491 | 0.4496 – 0.4829 |
|  | TAb (QUAL) | 1.1231 – 1.1537 | -0.5845 – -0.2532 | -1.0793 – -0.7386 | -1.1447 – -0.8071 | 0.8625 – 1.1272 | -0.5315 – -0.3419 |
| 95% Confidence Interval (CI) (t1) | IgG (Ind) | -0.6434 – -0.0109 | 0.9668 –1.1783 | -1.1679 – -1.1117 | -1.0579 – -0.7637 | 1.0859 – 1.1590 | 0.0431 – 0.8893 |
|  | TAb (QUAL) | 1.0670 – 1.1469 | -0.6083 – -0.2376 | -1.7783 – 0.0364 | -1.1351 – -0.7415 | 0.8929 – 1.1072 | -0.9425 – 0.1282 |
| Median Ab (t2) | IgG (Ind) | -0.6846 | -0.5628 | 0.6234 | 0.7505 | -0.4552 | 0.6812 |
|  | TAb (QUAL) | -0.6181 | -0.7016 | 0.9753 | 0.4204 | -0.3043 | 1.153 |
| Interquartile range (IQR) (t2) | IgG (Ind) | -0.8692 – -0.8692 | -0.6093 – -0.4595 | 0.5149 –0.7496 | -0.4746 – 0.9210 | -0.5125 – -0.3422 | 0.6665 – 0.6959 |
|  | TAb (QUAL) | -0.7400 – -0.5076 | -0.8490 – -0.5688 | 0.6811 – 1.0609 | -0.2900 – 0.6675 | -0.3514 – 0.2313 | 1.111 – 1.153 |
| 95% Confidence Interval (CI) (t2) | IgG (Ind) | -0.9313 – -0.5304 | -0.6304 – -0.2223 | 0.3921 – 0.8900 | -0.0649 – 0.7427 | -0.5956 – -0.3572 | 0.3078 – 1.0545 |
|  | TAb (QUAL) | -0.7149 – -0.4359 | -0.8549 – -0.5422 | -0.1536 – 1.8260 | -0.1588 – 0.5249 | -0.3153 – 0.1287 | 1.0054 – 1.2457 |
| Median Ab (t3) | IgG (Ind) | 1.1460 | -0.5917 | 0.5233 | 0.3818 | -0.6914 | -1.147 |
|  | TAb (QUAL) | -0.5356 | 1.135 | 0.0477 | 0.7210 | -0.8124 | -0.6278 |
| Interquartile range (IQR) (t3) | IgG (Ind) | 1.0929 –1.1526 | -0.6876 – -0.5448 | 0.3845 –0.6376 | 0.1426 – 1.1281 | -0.7840 – -0.6399 | -1.149 – -1.145 |
|  | TAb (QUAL) | -0.6429 – -0.3977 | 1.102 – 1.151 | -0.3222 – 0.3982 | 0.4822 – 1.0042 | -1.0939 – -0.7757 | -0.7696 – -0.6220 |
| 95% Confidence Interval (CI) (t3) | IgG (Ind) | 0.9211 –1.1950 | -0.7588 – -0.5336 | 0.2238 –0.7737 | 0.2948 – 0.8490 | -0.7953 – -0.4967 | -1.1971 – -1.0977 |
|  | TAb (QUAL) | -0.6581 – -0.4050 | 1.0890 – 1.1541 | -1.7553 – 1.8248 | 0.5838 – 0.9268 | -1.0234 – -0.7901 | -1.1337 – -0.3031 |
